# Vitexin attenuates chronic kidney disease by inhibiting renal tubular epithelial cell ferroptosis via NRF2 activation

**DOI:** 10.1186/s10020-023-00735-1

**Published:** 2023-10-27

**Authors:** Jiayu Song, Hongri Wang, Jingyi Sheng, Wen Zhang, Juan Lei, Weihua Gan, Fangfang Cai, Yunwen Yang

**Affiliations:** 1 Department of Pediatric Nephrology, The Second Affiliated Hospital of Nanjing Medical University, Nanjing, 210003 Jiangsu China; 2 Nanjing Key Laboratory of Pediatrics, Children’s Hospital of Nanjing Medical University, 72 Guangzhou Road, Nanjing, 210008 China; 3 Department of Nephrology, Affiliated Hospital of Integrated Traditional Chinese and Western Medicine, Nanjing University of Chinese Medicine, Nanjing, China; 4 School of Biopharmacy, China Pharmaceutical University, 639 Longmian Avenue, Nanjing, 211198 China

**Keywords:** Vitexin, Ferroptosis, CKD, NRF2, Antioxidant

## Abstract

**Background:**

Chronic kidney disease (CKD) involves a variety of pathological processes, and ferroptosis plays a vital role in CKD progression. Targeting ferroptosis is a promising strategy for the treatment of CKD. However, inhibitors of ferroptosis have not been used in the clinical treatment of CKD. Vitexin is a natural flavonoid with many biological activities and protective effects against various diseases. However, whether vitexin can prevent the progression of CKD is not known.

**Methods:**

In vivo, the effect of vitexin on CKD was evaluated by using mouse models of unilateral ureteral obstruction (UUO) and unilateral ischemia–reperfusion (UIR). Western blotting, Sirius red staining and transmission electron microscopy were used to analyze renal tubular injury, interstitial fibrosis, and inflammation in the kidneys of UUO and UIR mice. In vitro, CCK8 assays and lipid peroxidation assays were performed to analyze cell viability and lipid peroxidation in human renal tubular epithelial cells (HK2 cells) induced by erastin. The activation of renal fibroblasts (NRK-49 F cells) was also analyzed. Additionally, an in-silico protein-drug docking model and coimmunoprecipitation were performed to determine the direct substrate of vitexin.

**Results:**

In vivo, vitexin treatment significantly ameliorated renal tubular injury, interstitial fibrosis, and inflammation in the kidneys of UUO and UIR mice. Additionally, our results showed that vitexin significantly attenuated UUO- and UIR-induced ferroptosis in renal tubular epithelial cells by upregulating glutathione peroxidase 4 (GPX4) protein levels and inhibiting lipid peroxidation in mouse kidneys. In vitro, treatment with vitexin inhibited erastin-induced ferroptosis in HK2 cells. Moreover, vitexin inhibited the expression of collagen I and α-SMA (alpha-smooth muscle actin) in NRK-49 F cells induced by the supernatant of erastin-treated HK2 cells. Mechanistically, our results suggested that vitexin could activate the NRF2/heme oxygenase-1 (HO-1) pathway by inhibiting the KEAP1- and ubiquitination-mediated degradation of NRF2, thereby increasing the expression of GPX4, and further inhibiting lipid peroxidation and ferroptosis. Additionally, knockout of NRF2 greatly inhibited the antiferroptotic effects of vitexin.

**Conclusions:**

Taken together, our results indicate that vitexin can protect against renal tubular epithelial cell ferroptosis in CKD by activating the KEAP1/NRF2/HO-1 pathway and is a promising drug to treat CKD.

**Supplementary Information:**

The online version contains supplementary material available at 10.1186/s10020-023-00735-1.

## Background

Chronic kidney disease (CKD) is a complex life-threatening problem worldwide that affects nearly 13% of adults annually and increases social burdens (Evans et al. 2022). However, current clinical therapies only delay or prevent the progression of CKD to end-stage renal disease, which is lethal without renal replacement therapy. Thus, it is critical and urgent to develop powerful approaches to slow CKD. The ultimate pathophysiology of CKD is renal fibrosis, which is characterized by glomerulosclerosis, tubulointerstitial fibrosis, and inflammatory cell infiltration. During the development of tubulointerstitial fibrosis, the death of tubular epithelial cells (TECs) is considered a prominent event and inflammation may pre-exist the degenerative process (Djudjaj and Boor 2019). Persistent TEC death is typically accompanied by the abundant release of cytokines and inflammatory chemokines, which promote interstitial fibroblast proliferation (Humphreys 2018). In addition, epithelial-mesenchymal transition (EMT) and excessive extracellular matrix (ECM) production of collagen and fibronectin by renal myofibroblasts contribute to the development of fibrosis (Braga et al. 2022). Thus, directly targeting TEC death may be a therapeutic strategy for improving CKD.

Disturbances in iron and lipid metabolism have been reported in most CKD patients and animal models of CKD, ultimately resulting in ferroptosis in renal TECs (Wang et al. 2021; Naito et al. 2015). Ferroptosis is defined as an iron-dependent form of programmed cell death characterized by fatal lipid peroxidation with particular triggers, modulators, and effectors (Hirschhorn and Stockwell 2019). Nuclear factor-E2-related factor 2 (NRF2), which is an antioxidant transcription factor, directly or indirectly affects lipid-based reactive oxygen species (ROS) through different pathways. At baseline, cellular protein levels of NRF2 are low due to negative regulation by Kelch-like ECH-associated protein 1 (KEAP1), which is an essential substrate adaptor protein that binds to NRF2 and mediates the ubiquitination and proteasomal degradation of NRF2 (Pallesen et al. 2018). Under oxidative stress conditions, ROS perturb the KEAP1-NRF2 complex and prevent the ubiquitination of NRF2 (Motohashi et al. 2004). Consequently, a large amount of NRF2 translocates to the nucleus, inducing the expression of antioxidant enzymes (Magesh et al. 2012; Baird et al. 2013). Deletion or inhibition of NRF2 signaling involves glutathione (GSH) depletion, decreased activity of glutathione peroxidase 4 (GPX4) and iron-dependent accumulation of ROS, which ultimately lead to oxidative ferroptosis (Li et al. 2020). Studies have shown that ferroptosis is a new therapeutic target for CKD. In a 5/6 nephrectomy rat model of CKD, kidney injury was exacerbated by the ferroptosis inducer cisplatin, while the ferroptosis inhibitor deferoxamine mesylate (DFO) had antifibrotic effects (Wang et al. 2022). Additionally, treatment with DFO widely alleviated renal fibrosis and inflammatory cell infiltration in unilateral ureteral obstruction (UUO) or unilateral ischemia‒reperfusion (UIR) mouse models (Zhou et al. 2022). As shown by this evidence, the regulation of ferroptosis is critical in restoring renal function in CKD. However, no inhibitors of ferroptosis have been used in the clinic to treat CKD due to side effects. It is important to search for new inhibitors of ferroptosis to treat CKD.

Currently, some scholars have reported that natural flavonoids such as nobiletin show antiferroptotic, anti-inflammatory, and antifibrotic effects and prevent CKD progression (Lo et al. 2022). Vitexin (apigenin-8-C-glucoside) is a c-glycosylated flavonoid that is extracted from *Crataegus pinnatifida*, mung bean, passion flower and other natural plants (He et al. 2016). Vitexin is an active component of traditional Chinese medicines that has antioxidant, anticancer, anti-inflammatory, and immunomodulatory properties (Jiang et al. 2018; Zhao et al. 2021; Babaei et al. 2020; Chen et al. 2021). To date, no studies have clarified the effects of vitexin on the progression of CKD. Recently, Lei Guo et al. found that vitexin could attenuate oxidative injury and ferroptosis to protect against cerebral ischemiareperfusion injury (Guo and Shi 2022). However, whether vitexin exerts protective effects by inhibiting ferroptosis in CKD and the specific mechanism remain unknown.

This study investigated the underlying anti-inflammatory and antifibrotic roles and possible mechanisms of vitexin in the treatment of CKD. Our results suggested that vitexin was an alternative therapeutic candidate for treating CKD by targeting ferroptosis.

## Methods

### Vitexin

Vitexin (purity 99.90%) was obtained from MedChemExpress Limited Liability Company (HY-N0013, MCE, Shanghai, China). Vitexin is a c-glycosylated flavone compound extracted from Leguminosae, such as Trigonella foenum-graecum L. The formula of vitexin is C_21_H_20_O_10_, and it has a molecular weight of 432.38 g/mol. In this study, vitexin was dissolved in a solution with 10% DMSO + 40% PEG300 + 5% Tween-80 + 45% saline.

### Animal care, vitexin treatment and construction of the CKD mouse models

Male C57BL/6J mice (8 weeks of age, 25 g) were purchased from the Laboratory Animal Center of Nanjing Medical University (Nanjing, China). All animals were humanely housed with food and water ad libitum in a stable cage. Animal surgical preparations and treatments were performed according to the Animal Ethics Committee of Nanjing Medical University. In brief, we performed UUO and UIR surgery to establish CKD mouse models as previously described (Kramann and Menzel 2021). In the UUO experiment, after at least 7 days of acclimation, the mice were randomly divided into the vehicle sham, vehicle UUO, vitexin sham, and vitexin UUO groups (n = 6). Mice in the UUO groups were subjected to a surgical unilateral ureteral obstruction procedure to induce CKD. Due to the poor gastrointestinal bioavailability of vitexin in vivo, vitexin with a purity of 99.90% (MedChemExpress, HY-N0013) was dissolved in 10% DMSO + 40% PEG300 + 5% Tween-80 + 45% saline, and the mice were administered vitexin at a dose of 30 mg/kg/day body weight by gavage according to previous studies (Umar Ijaz et al. 2021) (Ding et al. 2021). Vitexin or vehicle was intragastrically administered daily after surgery. In the UIR experiment, four groups were established: the vehicle sham, vehicle UIR, vitexin sham, and vitexin UIR groups (n = 6). Mice in the UIR groups were subjected to CKD by surgical unilateral ischemia‒reperfusion. Vehicle or 30 mg/kg vitexin was intragastrically administered daily for 21 days. All mice in this study were sacrificed, and kidney samples were harvested and properly preserved for further analyses. Female hormones can affect fibrosis progression, resulting in variations. Therefore, only male mice were used in our animal studies.

### Cell culture

The HK2 and NRK-49 F cell lines were obtained from ATCC (Manassas, USA). HK2 cells were grown in DMEM/F-12 medium (319-075-CL, Gibco, USA) supplemented with FBS (26,170,035, Gibco, USA) at 37 °C with 5% CO_2_. To investigate the effect of vitexin, HK2 cells were exposed to certain concentrations of vitexin for 24 h. To induce ferroptosis, HK2 cells were pretreated with 100 µM vitexin for one hour and then incubated with the ferroptosis activator erastin (10 µM, 571203-78-6, MCE, Shanghai, China) for 24 h. HK2-conditioned medium was collected and used in coculture experiments with NRK-49 F cells. In the cell transfection experiment, the CRISPR/Cas9 plasmid targeting human NRF2 was synthesized by Tsingke Biotechnology (Nanjing, China), and the sequences are listed in the supplementary data. The NRF2-knockout plasmid and empty vector were transfected into HK2 cells with Lipofectamine (11,668,030, Thermo Fisher, USA). All cells were harvested for subsequent analysis.

### RNA isolation and quantitative real-time reverse transcription PCR (qRT‒PCR)

RNA was extracted from kidney tissues or cultured cells with TRIzol reagent (9108, Takara, Osaka, Japan) and reverse transcribed into cDNA using transcriptase reagents (2641 A, Takara, Osaka, Japan). QRT‒PCR was conducted using SYBR Green (q111-02/03, Vazyme, Nanjing, China) on an ABI detection system (CA, USA). All primer sequences are listed in the supplementary Table 1. MRNA expression was estimated by the cycle threshold values (ΔCt) and normalized to the housekeeping gene.

### Western blotting (WB) and coimmunoprecipitation (CO-IP)

Kidney tissues and HK2 and NRK-49 F cells were lysed with RIPA buffer (P0013B, Beyotime, Shanghai, China) containing a protease inhibitor cocktail (11,697,498,001, Roche, Indianapolis, USA). Nuclear and cytoplasmic fractions were isolated from fresh renal cortex tissue with a Nuclear/Cytoplasmic Protein Extraction Kit (P0028, Beyotime, Shanghai, China). Equal amounts of each sample were acquired, and WB was conducted according to conventional instructions. The primary and secondary antibodies are shown in the supplementary Table 2. The bound antibodies were visualized with an enhanced chemiluminescence detection system (Bio-Rad, CA, USA). Subsequently, the abundance of proteins was evaluated using ImageJ (NIH, USA). The co-IP assay was conducted with protein A/G beads (MedChemExpress, HY-K0202) as indicated by the instructions. In brief, cells were lysed in co-IP lysis buffer. The supernatant and NRF2 antibodies were incubated together and then centrifuged with protein A/G. After washing and eluting the sample, WB was performed to detect ubiquitin-linked NRF2.

### Periodic acid schiff (PAS) and sirius Red staining

Before proceeding with the staining protocol, the kidney was fixed in 4% paraformaldehyde, embedded in paraffin, and sectioned at a thickness of 4 μm with a slicer. After deparaffination and rehydration, the slides were stained with PAS (G1281, Solarbio, China) or Sirius Red (BA4079B, BASO, China). Images were captured at 200× magnification by an Olympus microscope (Tokyo, Japan). The renal injury index of pathological lesions in the PAS assay was scored according to previous reports (Weidemann et al. 2008). The relative Sirius Red-stained fibrotic area in the renal cortex was analyzed using a double-blind method (Turnberg et al. 2006).

### Immunofluorescence (IF) staining

For cellular IF analysis, NRK-49 F cells were seeded on round coverslips and treated. The coverslips were fixed in 10% formaldehyde at room temperature. For kidney IF analysis, mouse renal tissues were fixed in a formaldehyde solution and dehydrated in sucrose. After being permeabilized in 0.5% Triton X-100, the sections of cells or tissues were blocked with FBS. Afterward, the samples were incubated with primary and secondary antibodies (Supplementary Table 3). Finally, a fluorescence microscope (Carl Zeiss, Oberkochen, Germany) was used to observe the images.

### Immunohistochemical (IHC) staining

To perform IHC staining, the tissues were cut into 4 μm sections. The slides were deparaffinized and incubated with citrate buffer. After being treated with H_2_O_2_ to inhibit endogenous peroxidase, the sections were blocked with diluted BSA. The antibodies used for IHC staining are shown in the supplementary Table 4. Finally, IHC images were taken by a microscope (Olympus Co., Tokyo, Japan), and the positive fields were quantified with ImageJ.

### Transmission electron microscopy (TEM)

Renal sections were fixed in glutaraldehyde and then postfixed with 1% osmium tetraoxide. The samples were dehydrated using acetone and embedded in Spurr resin. The slices were cut by an ultramicrotome and sequentially stained with lead citrate and uranyl acetate. The ultrastructure of the kidney was imaged by a transmission electron microscope (JEOL JEM-1010, Tokyo, Japan). The percentage of damaged mitochondria was evaluated with ImageJ software as previously described (Song et al. 2022).

### TdT-mediated dUTP nick end labeling (TUNEL) assay

A BrightGreen kit (A112-01/02/03, Vazyme, Nanjing, China) was used according to the manufacturer’s instructions. Briefly, after reaction buffer containing TdT was applied to the renal sections, apoptotic cells were examined using a Carl Zeiss fluorescence microscope. At least five randomly selected visual areas per sample were imaged under a light microscope at 200× in a blinded manner. Finally, we calculated the positive fluorescent cell counts in the groups.

### Cell viability, cell proliferation and cytotoxicity assay

We performed a CCK8 assay (KGA317, KeyGen Biotech, Nanjing, China) to examine cell viability. HK2 cells were cultured in a plate and treated as described when the cell density reached an appropriate level. After adding CCK-8 reagent to each well, the cells were kept in the incubator for 1–4 h. The absorbance was measured with a customized microplate reader, and the values were calculated relative to the control. In addition, HK2 cell injury was measured by determining LDH levels in the supernatant with a biochemical analyzer (Hitachi Ltd., Tokyo, Japan). The proliferation of NRK-49 F cells was examined by a 5-ethynyl-2’-deoxyuridine (EdU) in vitro kit (C0071S, Beyotime, China) according to a standardized procedure. In brief, EdU reagent solution was added to the cells in the wells to bind to the newly synthesized DNA. Apollo green fluorescent dye was subsequently added to permanently label the newly divided cells, and then the cells were redyed with Hoechst. Photographs were obtained using fluorescence microscopy and used to calculate the percentage of fibrocyte proliferation.

### Determination of lipid peroxidation in kidney tissues and cells

Cellular lipid peroxide levels were assessed by BODIPY-C11 immunostaining. After living HK2 cells were seeded in laser confocal petri dishes, the cells were treated with erastin and/or vitexin. The dishes were removed from the incubator, and the cells were stained with the BODIPY-C11 581/591 sensor (D3861, Thermo Fisher, MA, USA) according to the manufacturer’s instructions. Different fluorescence emission peaks were observed by a Carl Zeiss confocal microscope. The levels of malondialdehyde (MDA), glutathione (GSH), and glutathione disulfide (GSSG) correlated with membrane lipid peroxidation during ferroptosis. Renal tissues or HK2 cells were homogenized in extraction buffer. MDA activity was detected with a customized commercial kit (S0131, Beyotime, Shanghai, China). The relative ratio of GSH/GSSG was assessed by a quantitative kit (S0053, Beyotime, Shanghai, China). The absorbances of MDA and GSH/GSSG were measured with an automated Bio-Rad microplate analyzer using a 96-well plate.

### Intracellular iron assay

Kidney tissues from the different groups were collected and homogenized in iron assay buffer. Total Fe^2+^ levels in tissues were detected by an iron assay kit (BC5410, Solarbio, China) according to the manufacturer’s instructions. We measured the absorbance at 520 nm using a common microplate analyzer, and iron levels are shown as µmol/mg.

### Statistical analysis

Comparisons between two groups were performed using unpaired Student’s t tests and multiple comparisons were performed using one- or two-way ANOVA with GraphPad Prism 6.0 Software (San Diego, CA, USA). The data are displayed as the mean ± standard deviation (S.D.) for each experimental group. Statistical significance was accepted when P values were less than 0.05.

## Results

### Vitexin treatment alleviated renal fibrosis in a UUO mouse model

**Fig. 1:**
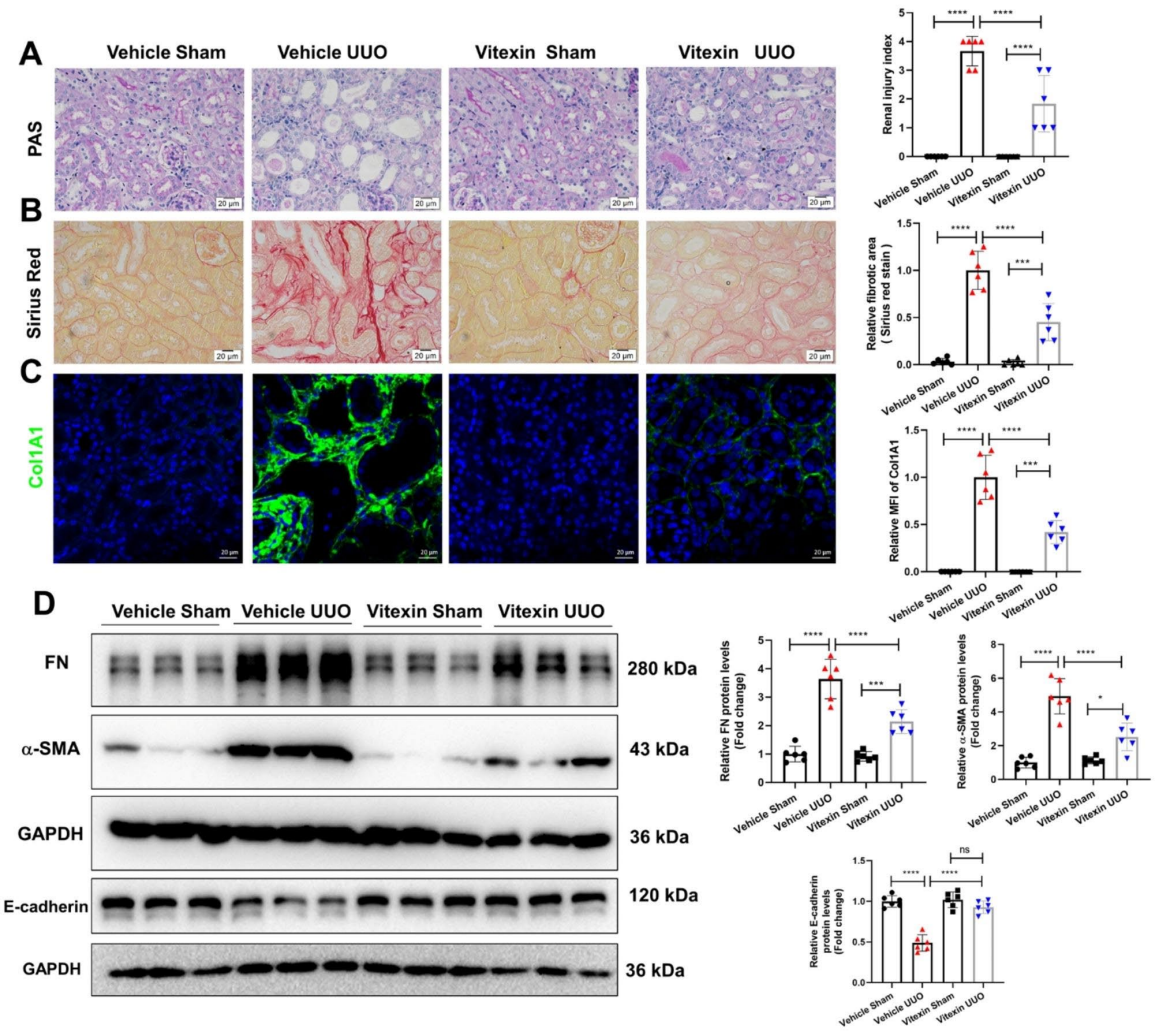
Vitexin alleviated renal fibrosis in the UUO mouse model. (**A**) PAS staining of kidney tissues and the renal injury index were calculated; magnification ×400; scale bar: 20 μm. Renal fibrosis was evaluated by (**B**) Sirius red and (**C**) IHC staining of collagen I. The images were quantified using ImageJ. (**D**) Representative protein expression of fibronectin, α-SMA and E-cadherin in the renal tissues of mice, as determined by Western blotting. Semiquantification of the blots is shown (right). The data are shown as the mean ± S.D. of each mouse (n = 6 mice in each group). ****P < 0·0001, ***P < 0·001, ns: no significance (analyzed by one-way ANOVA).

To identify whether vitexin treatment ameliorates kidney fibrosis, we established a UUO mouse model to mimic CKD in vivo. Compared with those in the sham group, the obstructed kidneys in the UUO group exhibited severe structural disorders, including tubular dilation and intratubular cast formation, as shown by PAS staining. However, the kidneys of UUO mice treated with vitexin exhibited significantly less tubular injury than the kidneys of UUO mice (Fig. 1A). Next, we examined ECM deposition by Sirius red staining. As renal fibrosis mainly occurs in tubulointerstitium, we analyzed the collagen deposition in the tubular area but not in the nephron. The results showed that ECM deposition was markedly induced in the kidneys of UUO mice but was significantly reduced after vitexin treatment (Fig. 1B). The expression of collagen 1A1 was analyzed by immunofluorescence and was not detected in the vehicle + sham or vitexin + sham groups, but it was present in the UUO group and was significantly decreased in the vitexin + UUO group (Fig. 1C). The protein levels of fibronectin and α-SMA in the kidney tissue of the UUO group were markedly higher than those of the sham group, and treatment with vitexin reduced the expression of fibronectin and α-SMA (Fig. 1D). Furthermore, UUO induced EMT was also reversed after vitexin treatment as the decreased protein levels of e-cadherin in the kidneys of UUO mice were restored in vitexin UUO mice (Fig. 1D). Additionally, vitexin (30 mg/kg/d) treatment had no obvious side effects on kidney function, as shown by the levels of blood urea nitrogen (BUN) and serum creatinine (Scr) in mice (Supplementary Fig. 1). These results indicated that vitexin treatment alleviated UUO-induced renal damage and fibrosis.

### Vitexin reduced UUO-induced renal tubular epithelial cell death and inflammation

**Fig. 2:**
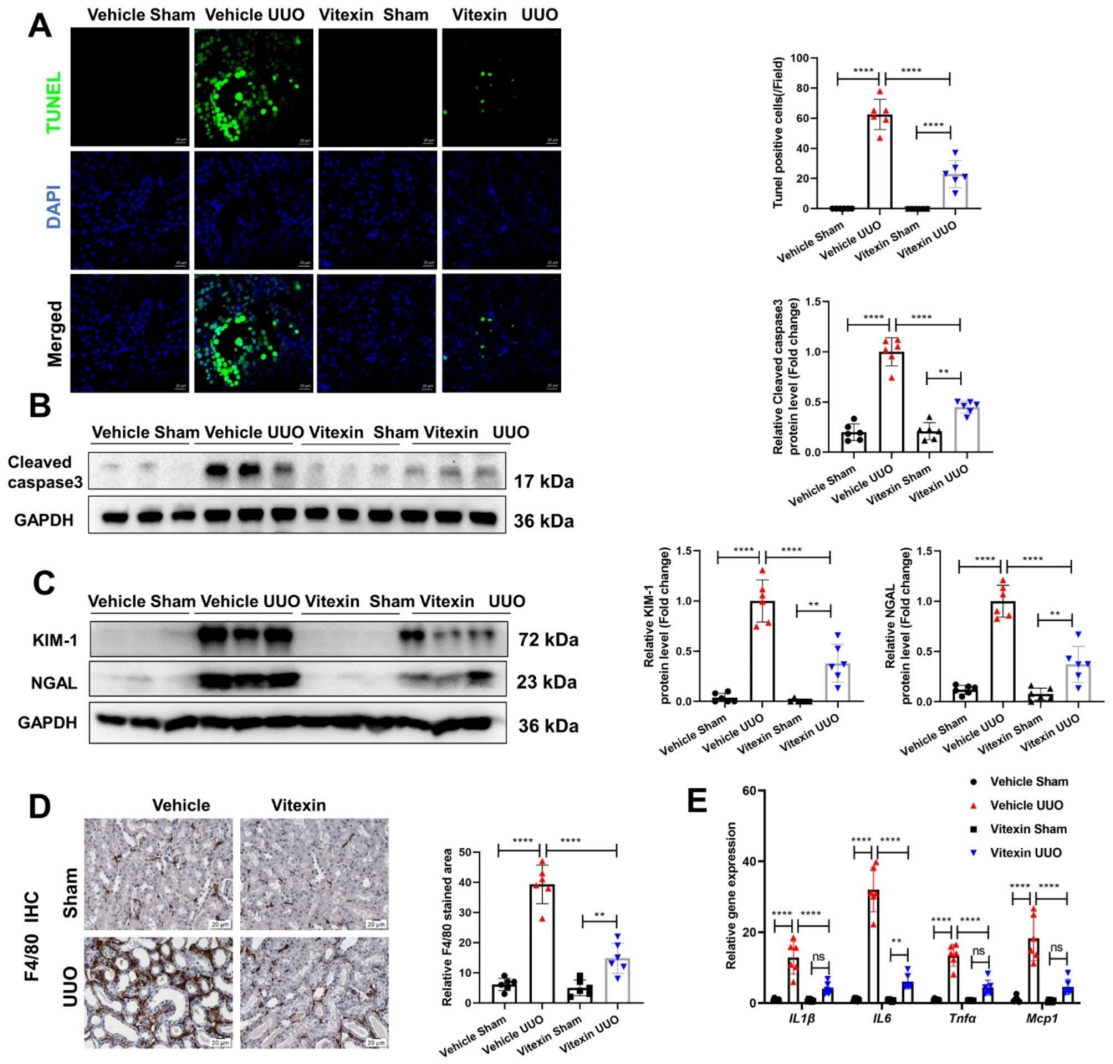
Vitexin reduced UUO-induced renal tubular epithelial cell death and inflammation. (**A**) Representative images of TUNEL staining in kidney tissues (magnification 400×; scale bar: 20 μm; green: TUNEL; blue: DAPI); the number of TUNEL-positive cells in each group was counted. (**B**) The protein levels of cleaved caspase3 in the kidneys of UUO mice with or without vitexin treatment were examined by WB; densitometry was performed by ImageJ, and the results are shown in the right panel. (**C**) The protein levels of KIM-1 and NGAL in the kidneys of UUO mice with or without vitexin treatment were examined by WB; densitometry was performed by ImageJ, and the results are shown in the right panel. (**D**) Representative IHC staining of F4/80 in the kidneys of UUO mice treated with or without vitexin (magnification 400×; scale bar: 20 μm) and the quantified results. (**E**) The mRNA levels of renal IL-1β, IL-6, TNF-α, and MCP-1 were analyzed by qRT‒PCR. The results are shown as the mean ± S.D. of 6 mice in each group. ****P < 0·0001, ***P < 0·001, ns: no significance (one-way ANOVA for A - D, two-way ANOVA for E)

Renal tubular epithelial cell death and the inflammatory response are key early events in CKD. TUNEL staining (Fig. 2A) and the protein levels of cleaved caspase3 (Fig. 2B) showed that cell death was increased in the kidneys of UUO mice but not in those of sham mice, while vitexin treatment inhibited cell death induced by UUO. As shown by the western blot (Fig. 2C) and qRT-PCR (Supplementary Fig. 2) results, vitexin treatment efficiently reversed the UUO-induced expression of the renal tubular injury indicators KIM-1 and NGAL. Inflammation is a hallmark of CKD and is characterized by inflammatory cell infiltration and inflammatory cytokine production. Immunohistochemical staining confirmed that the levels of F4/80-positive macrophages were elevated in the UUO group compared to the sham group. Vitexin significantly reduced F4/80-positive macrophages in UUO kidneys, indicating that vitexin effectively decreased macrophage infiltration (Fig. 2D). In addition, qRT‒PCR revealed that the mRNA levels of IL-1β, IL-6, TNF-α and MCP-1 were highly increased in the UUO group, while vitexin treatment abrogated the increase in these inflammatory cytokines (Fig. 2E). These results clearly demonstrated that vitexin could prevent UUO-induced renal cell death and inflammation in mice.

### Vitexin activated the KEAP1/NRF2/heme oxygenase-1 (HO-1) pathway in vivo and in vitro

**Fig. 3:**
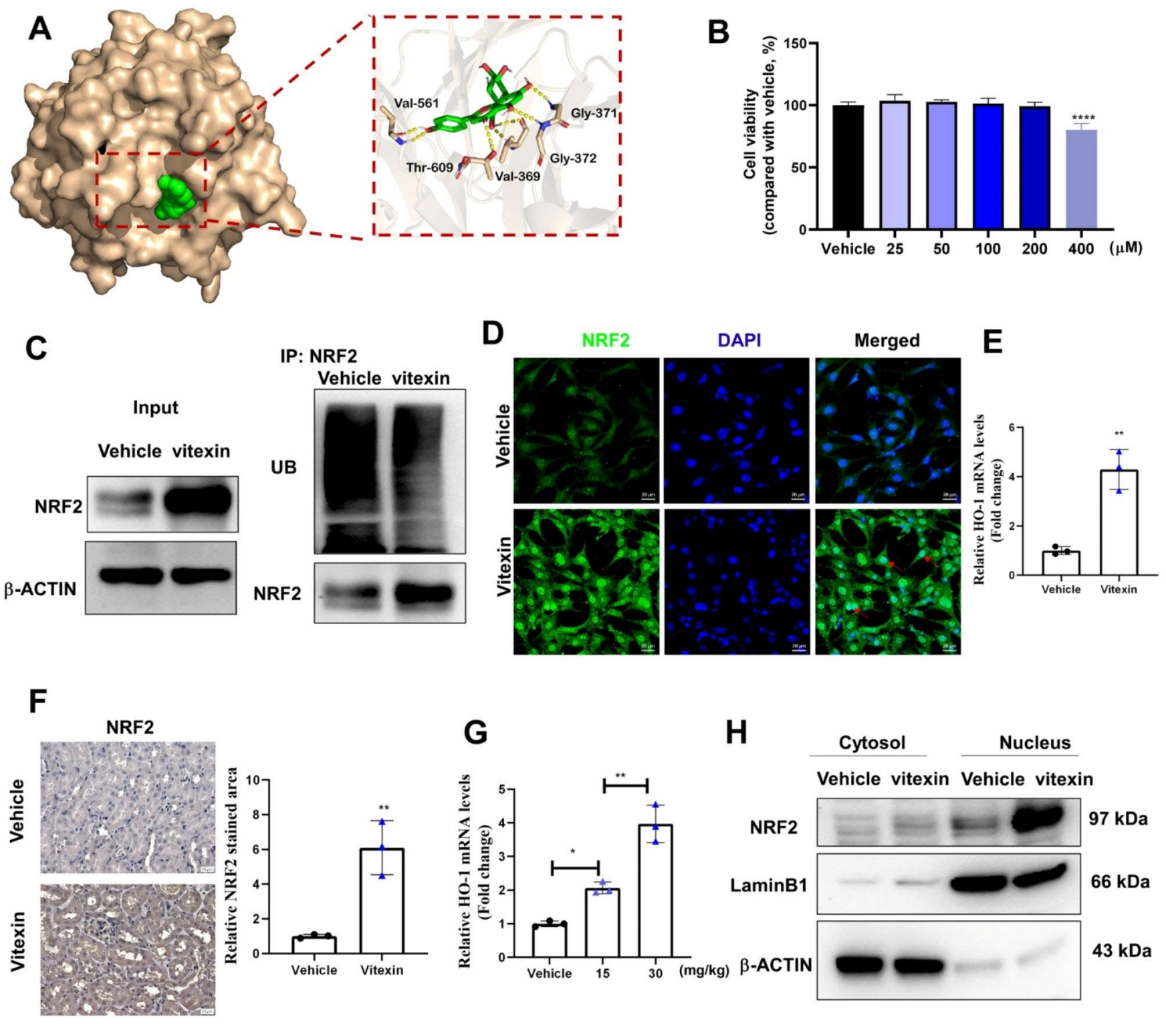
Vitexin regulated the expression of Keap1/NRF2/HO-1 in vivo and in vitro. (**A**) Predicted binding mode of vitexin with Keap1. (**B**) Viability of HK2 cells treated with vitexin. (**C**) Total NRF2 protein levels in HK2 cells pretreated with or without vitexin, as detected by WB. The ubiquitination of NRF2 was examined by immunoprecipitation assays. (**D**) Representative fluorescence images showing the colocalization of NRF2 and nuclei in HK2 cells incubated with vitexin (100 µmol/L). (**E**) Quantification of HO-1 mRNA expression in vitexin-treated HK2 cells. (**F**) Schematic IHC representation of NRF2 in vehicle- and vitexin-treated mouse kidney tissues and quantified images are shown. (**G**) Quantification of HO-1 mRNA expression in vitexin-treated mice kidneys. (**H**) NRF2 protein expression was measured by western blot. Lamin B was used as an internal control for nuclear proteins, and β-actin was used as an internal control for cytoplasmic proteins. The data are shown as the mean ± S.D. (n = 3). ****P < 0·0001, **P < 0·01, *P < 0·05 (*one-way ANOVA for B & G, t-text for E & F*)

Several studies have reported that vitexin exerts antioxidative effects by activating the NRF2 pathway; however, the precise mechanism is unclear (Guo and Shi 2023; Zhang et al. 2022). To mechanistically assess the protective effect of vitexin on CKD, we performed an in silico docking prediction using AutoDock (4.2.6) to identify the molecules that interacted with vitexin. First, molecular docking showed that vitexin could directly bind to KEAP1, which mediates the ubiquitination and degradation of NRF2 (Fig. 3A). As shown by CCK8 assays, vitexin (25 to 400 µM) had no toxic effect on the growth of HK2 cells compared with the vehicle. However, HK2 cell viability in the vitexin (400 µM) group was significantly lower than that in the other groups (Fig. 3B). When HK2 cells were treated with 100 µM vitexin, the ubiquitination-mediated degradation of NRF2 was inhibited (Fig. 3C). Moreover, vitexin markedly increased NRF2 translocation from the cytosol into the nucleus (indicated by red arrow), as shown by immunofluorescence staining (Fig. 3D). Furthermore, vitexin treatment robustly increased the mRNA levels of HO-1, which is a gene that is transcriptionally activated by NRF2, in HK2 cells (Fig. 3E). The immunohistochemical results showed that vitexin efficiently increased the levels of NRF2 in the renal tissues of mice (Fig. 3F). HO-1 levels in renal tissue were also observed after three days of intragastric vitexin administration. Intriguingly, pretreatment with 15 or 30 mg/kg vitexin upregulated HO-1 mRNA levels (Fig. 3G). Notably, the translocation of the NRF2 protein from the cytoplasm to the nucleus occurred in vitexin-treated renal tissues (Fig. 3H). The results in HK2 cells were consistent with those showing the changes in mouse kidney tissues. Therefore, these results show that KEAP1/NRF2/HO-1 is the main pathway affected by vitexin *in vivo and in vitro*.

### Vitexin inhibited renal tubular ferroptosis induced by UUO

**Fig. 4:**
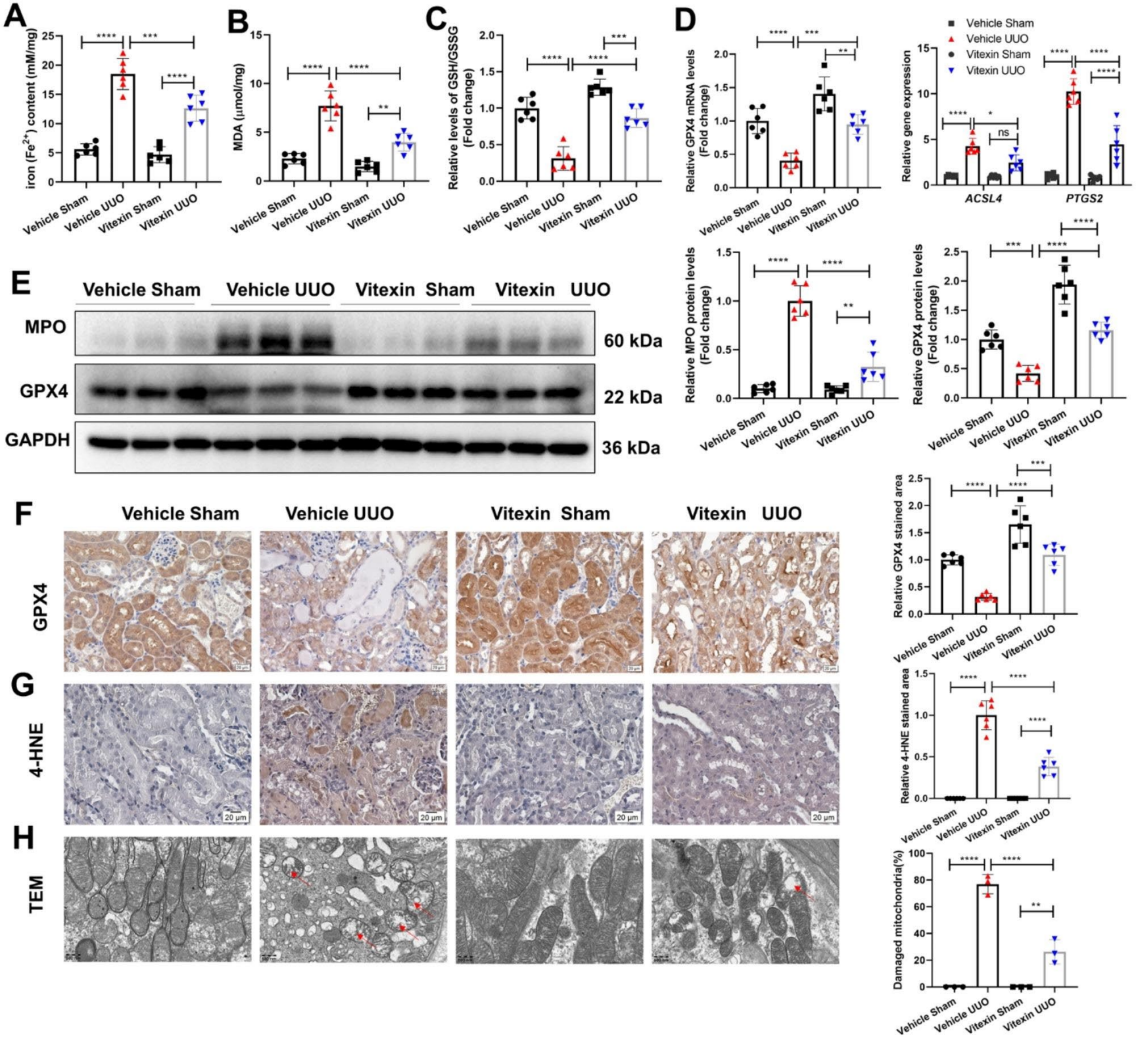
Vitexin inhibited renal tubular ferroptosis induced by UUO. (**A**) Iron levels (Fe^2+^) in the renal tissues of sham or UUO mice treated with vitexin. (**B**) The levels of MDA and (**C**) the GSH/GSSG ratio in UUO mice treated with or without vitexin. (**D**) Real-time quantitative PCR analysis was used to examine the expression of GPX4, ACSL4, and PTGS2 in the UUO-induced mouse model with or without vitexin treatment. (**E**) WB was performed to determine MPO and GPX4 protein levels, which were normalized to GAPDH and quantitatively analyzed by ImageJ software. (**F**) Renal slices from the different groups were collected for IHC staining of GPX4 and (**G**) 4-HNE. The relative stained area is shown in the histograms. (**H**) The percentages of damaged mitochondria are indicated by TEM images of renal tubular cells in the vitexin-treated UUO mouse model. All data are shown as the mean ± S.D (n = 3/6). ****P < 0·0001, ***P < 0·001, **P < 0·01 (one-way ANOVA for A-C & E-H & GPX4 in D, two-way ANOVA for D)

As discussed previously, NRF2 plays an important role in GPX4-dependent ferroptosis (Dodson et al. 2019), and we further examined whether vitexin inhibited GPX4-dependent ferroptosis through the NRF2 pathway. We measured the renal levels of chelatable iron (Fe^2+^). As expected, Fe^2+^ levels were enhanced in the UUO group compared with the sham group, and vitexin reduced these levels in UUO mice (Fig. 4A). MDA levels were lower in the UUO group than in the sham group, and vitexin increased these levels in the UUO group (Fig. 4B). Moreover, UUO induced a significant decrease in the GSSG/GSH ratio in renal tissues compared with that in the sham group, and vitexin treatment markedly increased this ratio in UUO mice (Fig. 4C). We also found that the mRNA level of GPX4 in the UUO group was lower than that in the sham group, and vitexin inhibited the downregulation of GPX4. Acyl-CoA synthetase long-chain family member 4 (ACSL4) and prostaglandin endoperoxide synthase 2 (PTGS2) expression was notably upregulated in the UUO group compared with the sham group after vitexin treatment (Fig. 4D). Additionally, the protein levels of myeloperoxidase (MPO) and GPX4 were detected by WB. We found that the UUO-induced upregulation of MPO and downregulation of GPX4 protein levels were greatly mitigated by vitexin (Fig. 4E). Next, we determined the levels of GPX4 and 4-hydroxynonenal (4-HNE) in renal tissues to assess lipid peroxidation-related ferroptosis. The IHC images showed that the decrease in GPX4 induced by UUO was abrogated by vitexin treatment (Fig. 4F). Vitexin restored 4-HNE expression, which was upregulated in the renal tubules of UUO mice (Fig. 4G). Figure 4 H shows the percentage of damaged mitochondria, which featured disrupted cristae, atrophy, and mitochondrial membrane rupture. We noted that mitochondrial damage did not occur in the sham group but was severe in the UUO group, and this effect was robustly alleviated by vitexin treatment. Additionally, whether vitexin treatment inhibited GPX4-independent ferroptosis induced by UUO was also analyzed. As the FSP1-CoQ10 and DHODH pathways play important roles in GPX4-independent ferroptosis (Doll et al. 2019; Mao et al. 2021), the protein levels of FSP1 and DHODH were analyzed in mouse kidneys after vitexin treatment. Our results showed that the decreased in protein levels of FSP1 and DHODH were almost not restored after vitexin treatment in the kidneys of UUO mice (Supplementary Fig. 3). These results suggested that vitexin inhibited GPX4-dependent ferroptosis. Overall, these findings demonstrated that vitexin could reduce the occurrence of TEC GPX4-dependent ferroptosis caused by UUO.

### Vitexin suppressed erastin-induced ferroptosis in renal tubular cells by activating NRF2

**Fig. 5:**
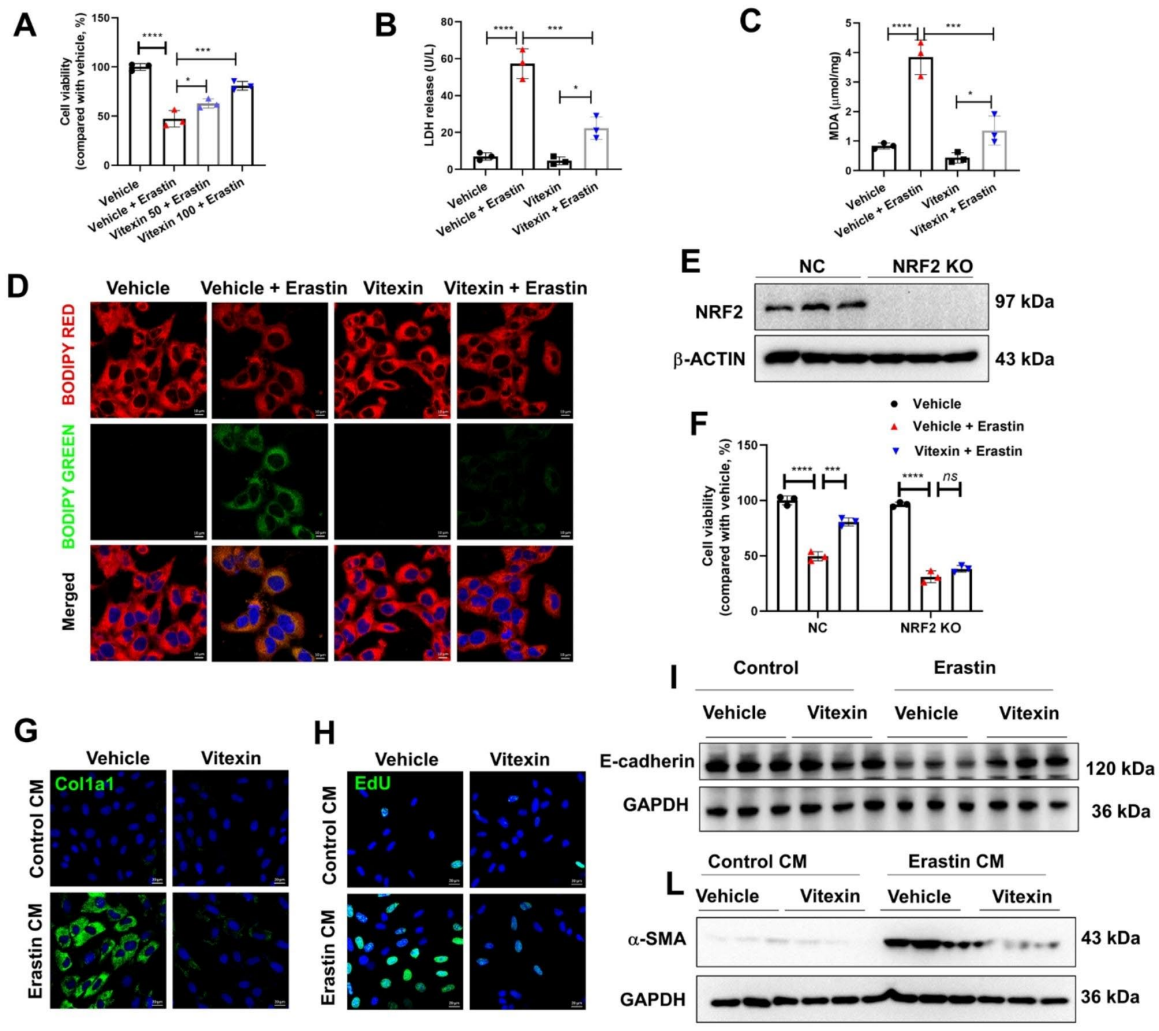
Vitexin suppressed erastin-induced ferroptosis by regulating NRF2. (**A**) CCK8 assays were used to assess the viability of HK2 cells exposed to vitexin (100 µM) and pretreated with or without erastin (10 µM). (**B**) LDH and (**C**) MDA levels in HK2 cells after treatment with vitexin under basal conditions or after erastin administration. (**D**) Fluorescence images showing lipid peroxidation stained by the BODIPY-C11 probe (green: oxidized lipids; red: lipids; and blue: Hoechst; scale bar: 10 μm). (**E**) Western blot analysis of the indicated proteins in NRF2 KO HK2 cells. (**F**) Cell viability was determined after NRF2 KO or NC HK2 cells treated with erastin and/or vitexin. (**G**) Fluorescence images of Col1a1 and (**H**) EdU in NRK-49 F cells that were incubated with HK2-CM (the supernatant of HK2 cells that were pretreated with erastin and/or vitexin, CM: conditioned medium), scale bar = 20 μm. (**I**) WB was performed to determine E-cadherin protein levels in HK2 cells treated with erastin and/or vitexin. (**L**) Western blot images showing the protein level of α-SMA in NRK-49 F cells treated with HK2-CM. GAPDH was used as a loading control. The results of three independent experiments are expressed as the mean ± S.D. ****P < 0·0001, ***P < 0·001 (one-way ANOVA for A-C and two-way ANOVA for F)

Erastin is a common ferroptosis activator (Zhao et al. 2020), and we treated HK2 cells with erastin and vitexin and examined cell viability. The results showed that erastin strongly suppressed cell viability, and this effect was rescued by vitexin treatment in a concentration-dependent manner compared to that in untreated cells (Fig. 5A). Additionally, the release rate of lactate dehydrogenase (LDH) and MDA levels in response to erastin were decreased when 100 µM vitexin was administered (Fig. 5B & C). As shown in Fig. 5D, compared to vehicle-treated cells, erastin-treated HK2 cells showed a shift in the fluorescence intensity of the lipid peroxidation indicator BODIPY-C11 from red to green, and this effect was reversed by vitexin treatment, indicating that vitexin inhibited erastin-induced lipid peroxidation. To determine whether vitexin suppressed erastin-induced ferroptosis by regulating NRF2, we successfully knocked out NRF2 in HK2 cells (Fig. 5E). Furthermore, we found that in the negative control (NC) group, erastin significantly reduced cell viability, and this effect could be rescued by vitexin. Compared with that in the NRF2-knockout (KO) group, cell viability was decreased after erastin treatment, but no change was observed after vitexin treatment (Fig. 5F). Therefore, NRF2 knockout in HK2 cells significantly blunted the protective effect of vitexin. To analyze ECM deposition and EMT, NRK-49 F cells were cultured with the supernatant of HK2 cells that were pretreated with erastin (10 µM) and/or vitexin (100 µM). The fluorescence intensity of collagen 1α in the erastin group was stronger than that in the control group and was decreased in the vitexin + erastin group (Fig. 5G). In addition, we performed an EdU assay to assess renal fibroblast proliferation. Consistently, the number of EdU-positive fibroblasts was significantly increased after treatment with the supernatant of erastin treated-HK2 cells compared to treatment with the supernatant of erastin + vitexin treated-HK2 cells (Fig. 5H). Moreover, erastin induced the decreased protein levels of e-cadherin were also restored after vitexin treatment in HK2 cells (Fig. 5I). Consistently, after treatment with the supernatant of erastin treated-HK2 cells induced the increased protein levels of α-SMA in NRK-49 F cells compared to treatment with the supernatant of erastin + vitexin treated-HK2 cells (Fig. 5L). As the culture medium of erastin treated-HK2 cells contained unknown components including oxidized lipids, the direct effects of vitexin on oxidized lipids induced NRK-49 F cells activation were also analyzed. Our results showed vitexin treatment didn’t inhibit ox-LDL (Oxidized Low-density lipoprotein) induced activation of NRK-49 F cells (Supplementary Fig. 4A). Additionally, knockdown of NRF2 in NRK-49 F cells didn’t blunt the protect effects of vitexin (Supplementary Fig. 4B-4D). Collectively, vitexin was able to protect against erastin-induced ferroptosis and suppress EMT by modulating NRF2 in renal tubular cells.

### Vitexin attenuated the progression of CKD in a UIR mouse model

**Fig. 6:**
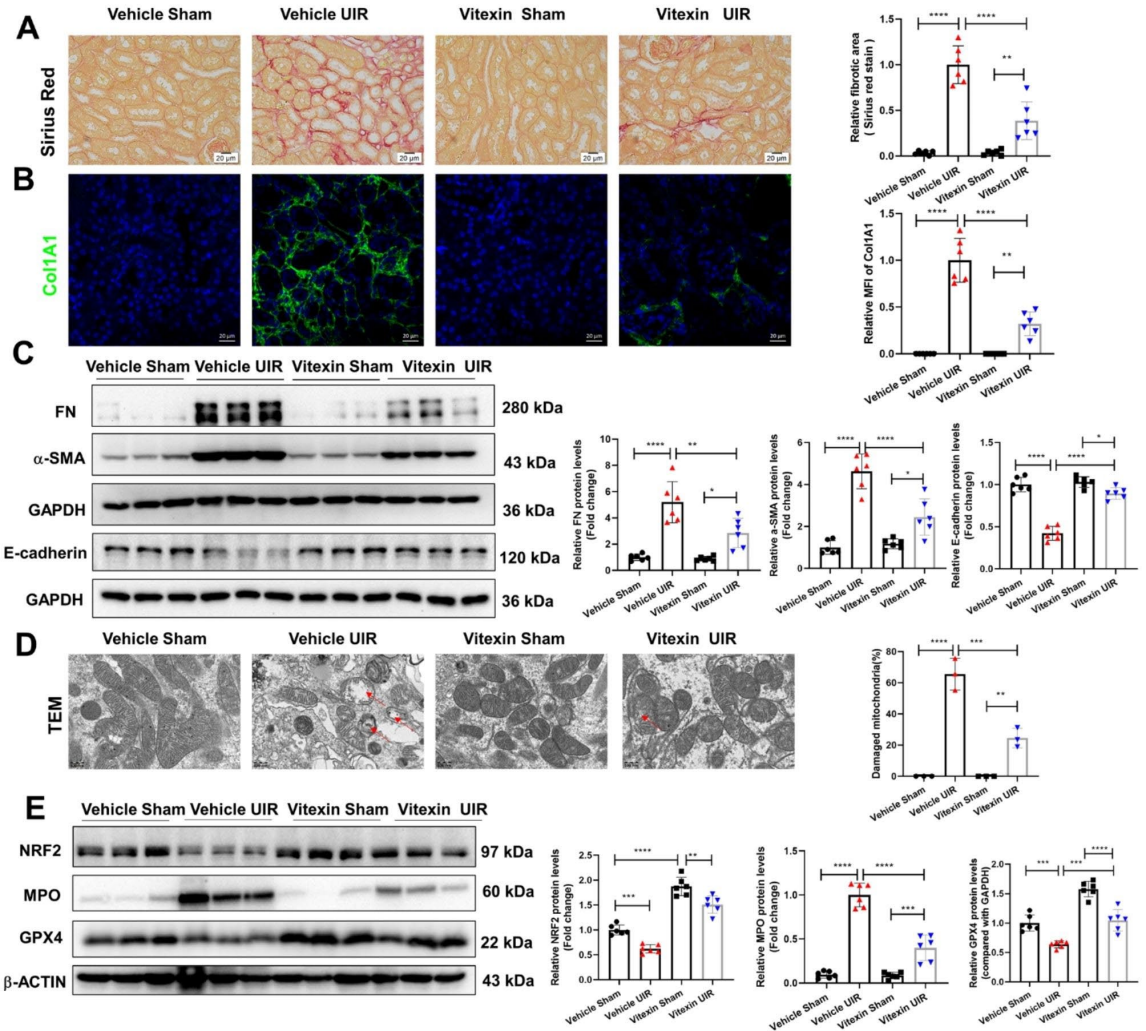
Vitexin treatment attenuates UIR-induced CKD in mice. (**A**) Sirius red staining of kidneys and relative fibrotic areas. (**B**) IHC staining of collagen I and the relative MFI of collagen I are shown on the right. (**C**) The protein expression of Fibronectin, α-SMA and E-cadherin in renal tissues was detected by WB and semiquantified. (**D**) Representative electron microscopy images and quantification of damaged mitochondria in renal tissues (scale bar: 1 μm). (**E**) The protein levels of NRF2, MPO and GPX4 in the kidneys of UIR mice with or without vitexin treatment were analyzed by WB, and the densitometry results are shown. The results are shown as the mean ± S.D. of 3 or 6 mice in each group; ****P < 0·0001, ***P < 0·001, **P < 0·01 (one-way ANOVA).

The unilateral ischemia‒reperfusion (UIR) mouse model is another stable and well-established model used to investigate the pathogenesis of CKD. We found that vitexin greatly mitigated collagen deposition around renal tubules caused by UIR (Fig. 6A & B). Vitexin substantially reduced the expression of fibronectin and α-SMA, which were strongly induced in the UIR group (Fig. 6C). Furthermore, UIR induced EMT was also reversed after vitexin treatment as the decreased protein levels of e-cadherin in the kidneys of UIR mice were restored in vitexin UIR mice (Fig. 6C). We also observed a lower percentage of damaged mitochondria in the vitexin + UIR group than in the UIR group (Fig. 6D). Moreover, vitexin effectively abrogated the decrease in NRF2 and GPX4 protein levels and notably abrogated the increase in MPO protein levels in the UIR group (Fig. 6E). Additionally, vitexin (30 mg/kg/d) treatment had no obvious side effects on kidney function, as shown by the levels of blood urea nitrogen (BUN) and serum creatinine (Scr) in mice (Supplementary Fig. 5). This observation proved that vitexin decreased kidney fibrosis and ameliorated ferroptosis-associated injury in UIR-induced CKD.

## Discussion

CKD is an ongoing destructive condition that has reached epidemic levels and leads to irreversible renal failure, ESRD and premature death. Causes contributing to CKD include renal parenchyma loss, chronic inflammation, fibrosis and reduced renal cellular regeneration (Ruiz-Ortega et al. 2020). The lack of satisfactory strategies to stop or reverse CKD progression underscores the need to develop effective targeted drugs. In this study, animals and cells were used to evaluate the renoprotective effects of vitexin on ferroptosis in CKD. To our knowledge, the present study is the first to examine the effect of vitexin on CKD.

Vitexin is a flavonoid isolated from natural sources and is used as a traditional Chinese medicine to treat a variety of illnesses, such as osteoarthritis (Yang et al. 2019), leukemia (Ling et al. 2018), stroke (Guo and Shi 2022), and various cancers (Ganesan and Xu 2017). As reported, vitexin protects against nephrolithiasis by inhibiting pyroptosis, apoptosis, EMT, and macrophage activation. Vitexin also protects against lipopolysaccharide (LPS)-induced apoptosis in rat kidney tubule epithelioid cells (Zeng et al. 2021). Vitexin was shown to attenuate mitochondrial dysfunction in rats with Cd-induced renal toxicity. In addition, vitexin was reported to inhibit the development of diabetic nephropathy by regulating NF-κB and AMPK signaling (Zhou et al. 2021). These results suggest promising roles for vitexin in improving kidney injury. In UUO- and UIR-induced CKD mouse models, we found that vitexin inhibited renal tubular cell injury, ECM deposition, EMT marker levels, and interstitial inflammation. Consistent with previous reports, our findings reveal that vitexin is an attractive candidate for treating CKD.

The ferroptotic death pathway involves pathological TEC death in a mouse model of UUO (Yang et al. 2020). A feature of ferroptosis is a nonapoptotic form of cell death characterized by mitochondrial contraction, increased membrane density, and mitochondrial cristae reduction (Gao et al. 2019). By examining mitochondrial morphology, the beneficial effects of vitexin on mitochondrial structural abnormities caused by UUO and UIR were observed. In addition, the death rate of TECs was high in the UUO mouse model, and vitexin markedly decreased this rate. We believe that TEC death might be related to ferroptosis. A large amount of evidence has demonstrated that robust accumulation of iron and lipid oxidative stress are the main characteristics of ferroptosis (Dixon et al. 2012). Iron levels, MDA, GSH/GSSG, ACSL4, PTGS2, MPO, 4-HNE and GPX4 are the major iron-related lipid peroxidation biomarkers. Therefore, we tested the levels of these biomarkers in cells and tissues to evaluate ferroptosis severity. Our results revealed that vitexin significantly increased GSH/GSSG, ACSL4, PTGS2 and GPX4 levels and decreased iron, MDA, MPO and 4-HNE levels induced by UUO or UIR. In vitro, vitexin increased cell viability and the levels of LDH and MDA in HK2 cells induced by the ferroptosis activator erastin and inhibited the activation of NRK-49 F cells. Overall, vitexin inhibited ferroptosis during the pathological processes of CKD.

Previous studies have suggested that vitexin exerts antioxidative effects by upregulating the NRF2 pathway in acute lung injury or cerebral injury (Guo and Shi 2022; Lu et al. 2018). NRF2 has attracted attention as a master antioxidative moderator that meditates oxidative damage and inflammation in CKD (Ito et al. 2020). NRF2 activation is controlled by the homeostasis of redox maintenance enzymes, including KEAP1 and HO-1 (Saha et al. 2020). In addition, NRF2 is an antioxidant transcription factor that regulates the expression of GPX4. GPX4 is an antioxidant defense enzyme that regulates ferroptosis by suppressing phospholipid peroxidation (Cui et al. 2022). In an LPS-induced acute kidney injury model, KEAP1-mediated NRF2/antioxidant response element system activation was critical in alleviating oxidative stress and ferroptosis (Qiu et al. 2022). In our study, molecular docking showed that vitexin directly bound to KEAP1, significantly increased NRF2 translocation from the cytosol into the nucleus and mediated KEAP1/NRF2/HO-1 signaling. Interestingly, GPX4 protein levels were upregulated by vitexin treatment in UUO/UIR model mice. These findings suggested that vitexin was necessary for restoring ROS metabolism and alleviating ferroptosis in CKD via the NRF2 pathway. However, evidence of a direct interaction between vitexin and KEAP1 was not validated in our study, although ubiquitination-mediated degradation of NRF2 was inhibited by vitexin. Additionally, other forms of regulated necrosis, such as pyroptosis, were not analyzed in this study. Although vitexin (30 mg/kg/d) treatment markedly protected against CKD progression, the bioavailability of vitexin in vivo was not analyzed. All this needs further research.

## Conclusion

**Fig. 7:**
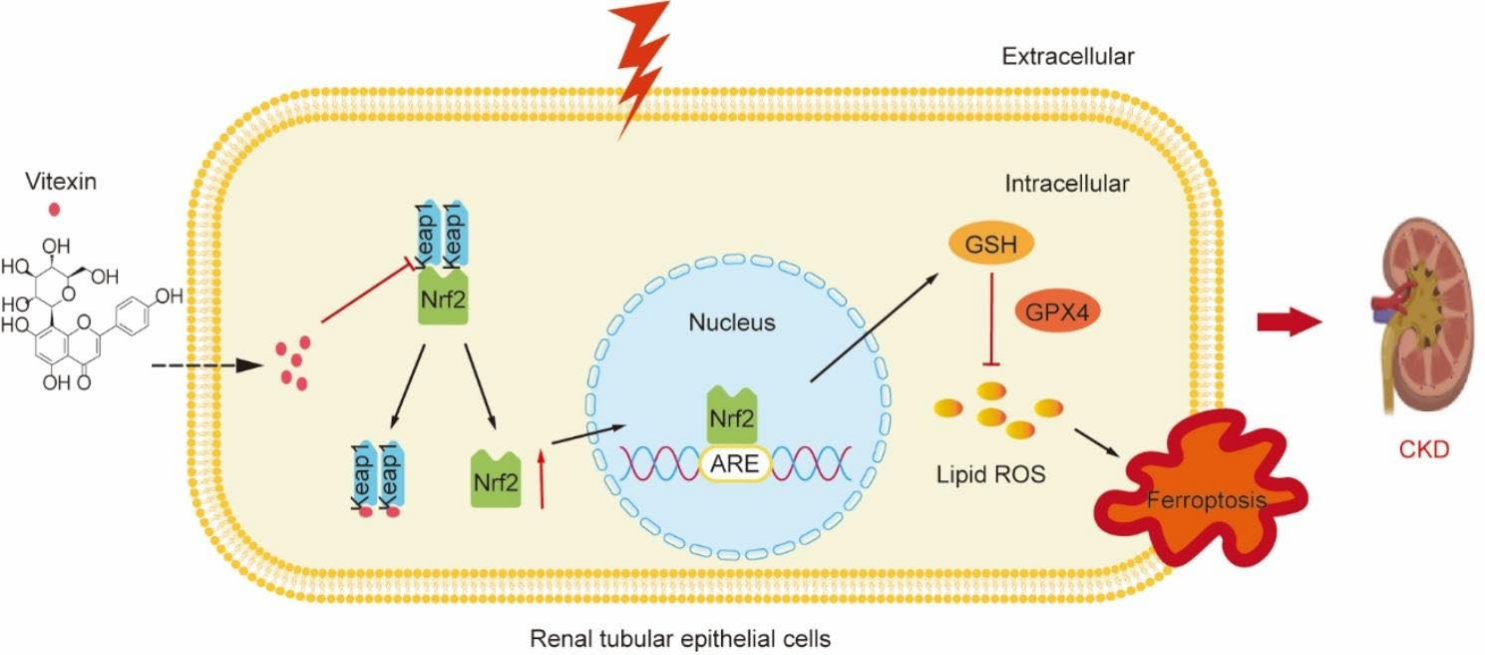
Research diagram. Mechanism by which vitexin inhibits ferroptosis and ameliorates chronic kidney disease

In summary, our data putatively suggest that vitexin protects against UUO/UIR-induced CKD. Furthermore, we demonstrated that vitexin exerts renoprotective effects by inhibiting ferroptosis by regulating the KEAP1/NRF2/HO-1 pathway and reducing GPX4-related lipid peroxidation (Fig. 7). This work demonstrated the efficacy and mechanisms of vitexin in animals and cells and identified a possible agent for the clinical treatment of CKD.

### Electronic supplementary material

Below is the link to the electronic supplementary material.

Supplementary Material 1

Supplementary Material 2

Supplementary Material 3

Supplementary Material 4

Supplementary Material 5

Supplementary Material 6

Supplementary Material 7

## Data Availability

The datasets used and/or analyzed during the current study are available from the corresponding author upon reasonable request.

## Abbreviations

CKD: Chronic kidney disease
UUO: Unilateral ureteral obstruction
UIR: Unilateral ischemia–reperfusion
TECs: Tubular epithelial cells
ECM: Extracellular matrix
EMT: Epithelial-Mesenchymal Transition
NRF2: Nuclear factor-E2-related factor 2
ROS: Reactive oxygen species
WB: Western blotting
PAS: Periodic acid-Schiff
IHC: Immunohistochemistry
TEM: Transmission electron microscopy
TUNEL: TdT-mediated dUTP nick end labeling
IF: Immunofluorescence
MDA: Malondialdehyde
4-HNE: 4-hydroxynonenal

## References

[CR19] Babaei F, Moafizad A, Darvishvand Z, Mirzababaei M, Hosseinzadeh H, Nassiri-Asl M (2020). Review of the effects of vitexin in oxidative stress-related diseases. Food Sci Nutr.

[CR11] Baird L, Lleres D, Swift S, Dinkova-Kostova AT (2013). Regulatory flexibility in the Nrf2-mediated stress response is conferred by conformational cycling of the Keap1-Nrf2 protein complex. Proc Natl Acad Sci U S A.

[CR4] Braga PC, Alves MG, Rodrigues AS, Oliveira PF. Mitochondrial pathophysiology on chronic kidney disease. Int J Mol Sci. 2022;23(3). 10.3390/ijms23031776 PMC8836100 35163697

[CR20] Chen Y, Wang B, Yuan X, Lu Y, Hu J, Gao J (2021). Vitexin prevents colitis-associated carcinogenesis in mice through regulating macrophage polarization. Phytomedicine.

[CR48] Cui C, Yang F, Li Q (2022). Post-translational modification of GPX4 is a Promising Target for treating ferroptosis-related Diseases. Front Mol Biosci.

[CR24] Ding T, Zhao T, Li Y, Liu Z, Ding J, Ji B (2021). Vitexin exerts protective effects against calcium oxalate crystal-induced kidney pyroptosis in vivo and in vitro. Phytomedicine.

[CR44] Dixon SJ, Lemberg KM, Lamprecht MR, Skouta R, Zaitsev EM, Gleason CE (2012). Ferroptosis: an iron-dependent form of nonapoptotic cell death. Cell.

[CR2] Djudjaj S, Boor P (2019). Cellular and molecular mechanisms of kidney fibrosis. Mol Aspects Med.

[CR30] Dodson M, Castro-Portuguez R, Zhang DD (2019). NRF2 plays a critical role in mitigating lipid peroxidation and ferroptosis. Redox Biol.

[CR31] Doll S, Freitas FP, Shah R, Aldrovandi M, da Silva MC, Ingold I (2019). FSP1 is a glutathione-independent ferroptosis suppressor. Nature.

[CR1] Evans M, Lewis RD, Morgan AR, Whyte MB, Hanif W, Bain SC (2022). A narrative review of chronic kidney disease in clinical practice: current Challenges and Future Perspectives. Adv Ther.

[CR37] Ganesan K, Xu B (2017). Molecular targets of vitexin and isovitexin in cancer therapy: a critical review. Ann N Y Acad Sci.

[CR43] Gao M, Yi J, Zhu J, Minikes AM, Monian P, Thompson CB et al. Role of Mitochondria in Ferroptosis. Mol Cell. 2019;73(2). 10.1016/j.molcel.2018.10.042 PMC6338496 30581146

[CR21] Guo L, Shi L. Vitexin improves cerebral ischemia–reperfusion Injury by attenuating oxidative Injury and Ferroptosis via Keap1/Nrf2/HO-1signaling. Neurochem Res. 2022. 10.1007/s11064-022-03829-0 36435955

[CR28] Guo L, Shi L (2023). Vitexin improves cerebral ischemia–reperfusion Injury by attenuating oxidative Injury and Ferroptosis via Keap1/Nrf2/HO-1signaling. Neurochem Res.

[CR16] He M, Min J-W, Kong W-L, He X-H, Li J-X (2016). Peng B-W. A review on the pharmacological effects of vitexin and isovitexin. Fitoterapia.

[CR7] Hirschhorn T, Stockwell BR (2019). The development of the concept of ferroptosis. Free Radic Biol Med.

[CR3] Humphreys BD (2018). Mechanisms of Renal Fibrosis. Annu Rev Physiol.

[CR46] Ito M, Tanaka T, Nangaku M (2020). Nuclear factor erythroid 2-related factor 2 as a treatment target of kidney diseases. Curr Opin Nephrol Hypertens.

[CR17] Jiang J, Dai J, Cui H (2018). Vitexin reverses the autophagy dysfunction to attenuate MCAO-induced cerebral ischemic stroke via mTOR/Ulk1 pathway. Biomed Pharmacother.

[CR22] Kramann R, Menzel S (2021). Mouse models of kidney fibrosis. Methods Mol Biol.

[CR12] Li J, Cao F, Yin H-L, Huang Z-J, Lin Z-T, Mao N (2020). Ferroptosis: past, present and future. Cell Death Dis.

[CR36] Ling T, Lang W, Feng X, Das S, Maier J, Jeffries C (2018). Novel vitexin-inspired scaffold against leukemia. Eur J Med Chem.

[CR15] Lo Y-H, Yang S-F, Cheng C-C, Hsu K-C, Chen Y-S, Chen Y-Y et al. Nobiletin alleviates Ferroptosis-Associated Renal Injury, inflammation, and fibrosis in a unilateral ureteral obstruction mouse model. Biomedicines. 2022;10(3). 10.3390/biomedicines10030595 PMC8944974 35327397

[CR45] Lu Y, Yu T, Liu J, Gu L (2018). Vitexin attenuates lipopolysaccharide-induced acute lung injury by controlling the Nrf2 pathway. PLoS ONE.

[CR10] Magesh S, Chen Y, Hu L (2012). Small molecule modulators of Keap1-Nrf2-ARE pathway as potential preventive and therapeutic agents. Med Res Rev.

[CR32] Mao C, Liu X, Zhang Y, Lei G, Yan Y, Lee H (2021). DHODH-mediated ferroptosis defence is a targetable vulnerability in cancer. Nature.

[CR9] Motohashi H, Katsuoka F, Engel JD, Yamamoto M (2004). Small maf proteins serve as transcriptional cofactors for keratinocyte differentiation in the Keap1-Nrf2 regulatory pathway. Proc Natl Acad Sci U S A.

[CR6] Naito Y, Fujii A, Sawada H, Oboshi M, Iwasaku T, Okuhara Y (2015). Association between renal iron accumulation and renal interstitial fibrosis in a rat model of chronic kidney disease. Hypertens Res.

[CR8] Pallesen JS, Tran KT, Bach A (2018). Non-covalent Small-Molecule Kelch-like ECH-Associated protein 1-Nuclear factor erythroid 2-Related factor 2 (Keap1-Nrf2) inhibitors and their potential for Targeting Central Nervous System Diseases. J Med Chem.

[CR49] Qiu W, Zhang X, Pang X, Huang J, Zhou S, Wang R (2022). Asiatic acid alleviates LPS-induced acute kidney injury in broilers by inhibiting oxidative stress and ferroptosis via activation of the Nrf2 pathway. Food Chem Toxicol.

[CR34] Ruiz-Ortega M, Rayego-Mateos S, Lamas S, Ortiz A, Rodrigues-Diez RR (2020). Targeting the progression of chronic kidney disease. Nat Rev Nephrol.

[CR47] Saha S, Buttari B, Panieri E, Profumo E, Saso L. An overview of Nrf2 Signaling Pathway and its role in inflammation. Molecules. 2020;25(22). 10.3390/molecules25225474 PMC7700122 33238435

[CR27] Song J, Sheng J, Lei J, Gan W, Yang Y (2022). Mitochondrial targeted antioxidant SKQ1 ameliorates acute kidney Injury by inhibiting ferroptosis. Oxidative Med Cell Longev.

[CR26] Turnberg D, Lewis M, Moss J, Xu Y, Botto M, Cook HT (2006). Complement activation contributes to both glomerular and tubulointerstitial damage in adriamycin nephropathy in mice. J Immunol.

[CR23] Umar Ijaz M, Batool M, Batool A, Al-Ghanimd KA, Zafar S, Ashraf A (2021). Protective effects of vitexin on cadmium-induced renal toxicity in rats. Saudi J Biol Sci.

[CR5] Wang J, Liu Y, Wang Y, Sun L (2021). The cross-link between ferroptosis and kidney Diseases. Oxid Med Cell Longev.

[CR13] Wang J, Wang Y, Liu Y, Cai X, Huang X, Fu W (2022). Ferroptosis, a new target for treatment of renal injury and fibrosis in a 5/6 nephrectomy-induced CKD rat model. Cell Death Discov.

[CR25] Weidemann A, Bernhardt WM, Klanke B, Daniel C, Buchholz B, Câmpean V (2008). HIF activation protects from acute kidney injury. J Am Soc Nephrol.

[CR35] Yang H, Huang J, Mao Y, Wang L, Li R, Ha C (2019). Vitexin alleviates interleukin-1β-induced inflammatory responses in chondrocytes from osteoarthritis patients: involvement of HIF-1α pathway. Scand J Immunol.

[CR42] Yang L, Guo J, Yu N, Liu Y, Song H, Niu J (2020). Tocilizumab mimotope alleviates kidney injury and fibrosis by inhibiting IL-6 signaling and ferroptosis in UUO model. Life Sci.

[CR39] Zeng M, Qi M, Kan Y, Zheng X, Feng W. A new flavonoid from the thorn of Lam. Nat Prod Res. 2021:1–6. 10.1080/14786419.2021.1969569 34448432

[CR29] Zhang S, Jin S, Zhang S, Li YY, Wang H, Chen Y et al. Vitexin protects against high glucose-induced endothelial cell apoptosis and oxidative stress via Wnt/beta-catenin and Nrf2 signalling pathway. Arch Physiol Biochem. 2022:1–10. 10.1080/13813455.2022.2028845 35254859

[CR33] Zhao Y, Li Y, Zhang R, Wang F, Wang T, Jiao Y (2020). The role of Erastin in ferroptosis and its prospects in Cancer Therapy. Onco Targets Ther.

[CR18] Zhao C-R, Yang F-F, Cui Q, Wang D, Zhou Y, Li Y-S (2021). Vitexin inhibits APEX1 to counteract the flow-induced endothelial inflammation. Proc Natl Acad Sci U S A.

[CR41] Zhou G, Cui J, Xie S, Wan H, Luo Y, Guo G (2021). Vitexin, a fenugreek glycoside, ameliorated obesity-induced diabetic nephropathy via modulation of NF-κB/IkBα and AMPK/ACC pathways in mice. Biosci Biotechnol Biochem.

[CR14] Zhou L, Xue X, Hou Q, Dai C (2022). Targeting ferroptosis attenuates interstitial inflammation and kidney fibrosis. Kidney Dis (Basel).

