# Supplementary material for: Vitexin attenuates chronic kidney disease by inhibiting renal tubular epithelial cell ferroptosis via NRF2 activation
Source: Mol Med. 2023 Oct 27;29:147. doi: 10.1186/s10020-023-00735-1 (PMC10612207; doi:10.1186/s10020-023-00735-1)
Supplement: Supplementary file 1 — Supplementary Material 1 [file 10020_2023_735_MOESM1_ESM.pdf]

SPRINGER NATURE

Author Services

Editing Certificate

This document certifies that the manuscript

Vitexin attenuates chronic kidney disease by inhibition of renal tubular epithelial cell ferroptosis via activating NRF2

prepared by the authors

Jiayu Song 1, Hongri Wang 1, Jingyi Sheng 1, Wen Zhang 3, Juan Lei 1, Weihua Gan 1*, Fangfang Cai 4*, Yunwen Yang 2*

was edited for proper English language, grammar, punctuation, spelling, and overall style by one or more of the highly qualified native English speaking editors at SNAS.

This certificate was issued on **August 17, 2023** and may be verified on the SNAS website using the verification code **ABD1-ACB5-E119-4F50-4A47**.

Neither the research content nor the authors' intentions were altered in any way during the editing process. Documents receiving this certification should be English-ready for publication; however, the author has the ability to accept or reject our suggestions and changes. To verify the final SNAS edited version, please visit our verification page at secure.authorservices.springernature.com/certificate/verify.
If you have any questions or concerns about this edited document, please contact SNAS at support@as.springernature.com.

SNAS provides a range of editing, translation, and manuscript services for researchers and publishers around the world.
For more information about our company, services, and partner discounts, please visit authorservices.springernature.com.
