# Supplementary material for: Vitexin attenuates chronic kidney disease by inhibiting renal tubular epithelial cell ferroptosis via NRF2 activation
Source: Mol Med. 2023 Oct 27;29:147. doi: 10.1186/s10020-023-00735-1 (PMC10612207; doi:10.1186/s10020-023-00735-1)
Supplement: Supplementary file 3 — Supplementary Material 3 [file 10020_2023_735_MOESM3_ESM.pdf]

AAGGAGTTTTGAGTCGTGGGGTAGGAAAAAAGATTTGTTATTTACAAACGGGGTCATGACTGGTTAGTAAG
TAGAGAGACACAGAACTGCAGCTGATTCCATTTTGTTTTGTAGTGGTGCCTTAGAGCTTACTCATCCCCTGTT
GGTGGAAGACTCATAAATCAATGCCTTATCAATTTTAGGTTTCTTCGGCTACGTTTCAGTCACTTGTTCCTGAT
ATTCCCGGTCACATCGAGAGCCCAGTCTTCATTGCTACTAATCAGGCTCAGTCACCTGAAACTTCTGTTGCTC
AGGTAGCCCCTGTTGATTTAGACGGTATGCAACAGGACATTGAGCAAGTTTGGGAGGAGCTATTATCCATTC
CTGAGTTACAGGTAACTAAAATAGAATGTAATACTGGAGATTTTTTTTATATTCAGTGCCTTTAGTCATTCTGAT
TATTTATATACCACCTATTTATAGGAAGGATTGGAGGGGTGCTATTAA
