# Supplementary figures and images for: Vitexin attenuates chronic kidney disease by inhibiting renal tubular epithelial cell ferroptosis via NRF2 activation
Source: Mol Med. 2023 Oct 27;29:147. doi: 10.1186/s10020-023-00735-1 (PMC10612207; doi:10.1186/s10020-023-00735-1)

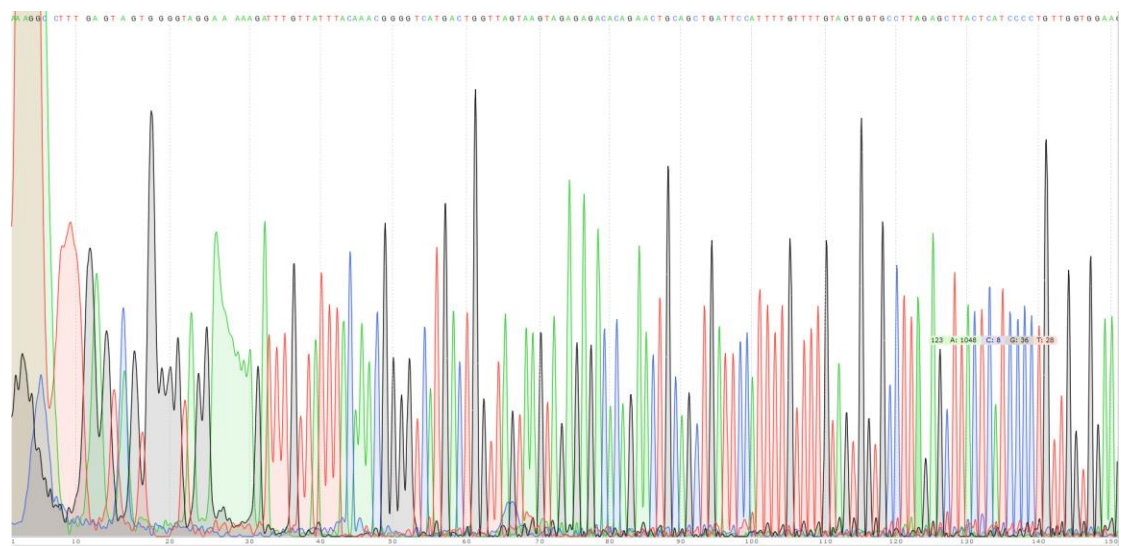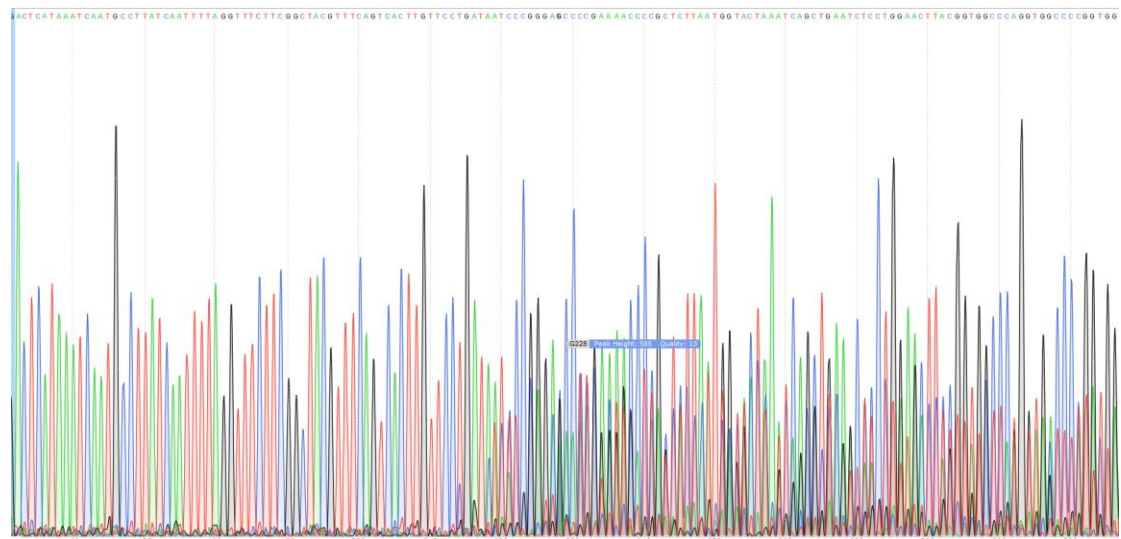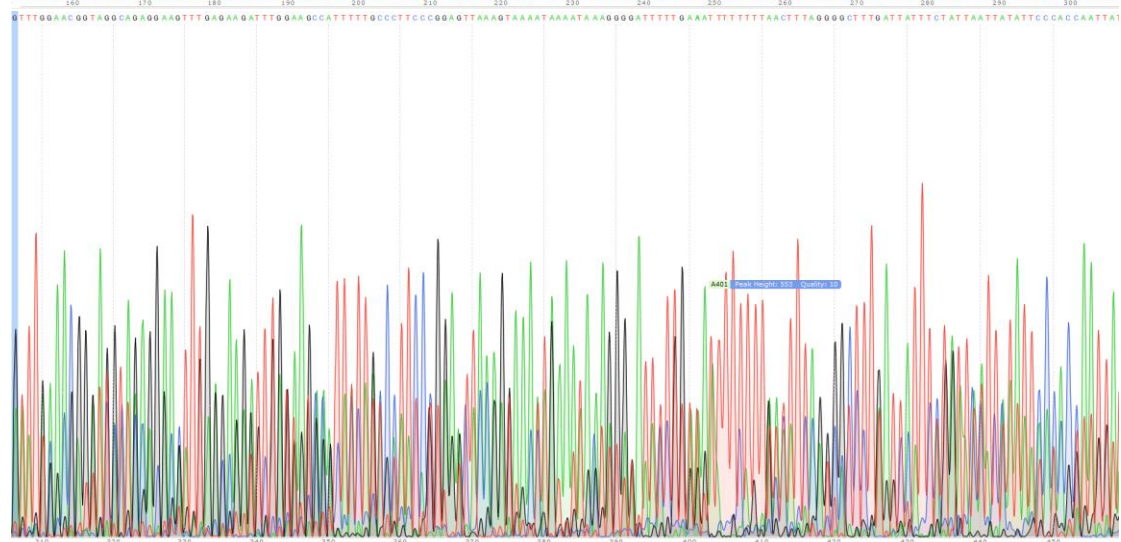

Supplement: Supplementary file 4 — Supplementary Material 4 [file 10020_2023_735_MOESM4_ESM.pdf]

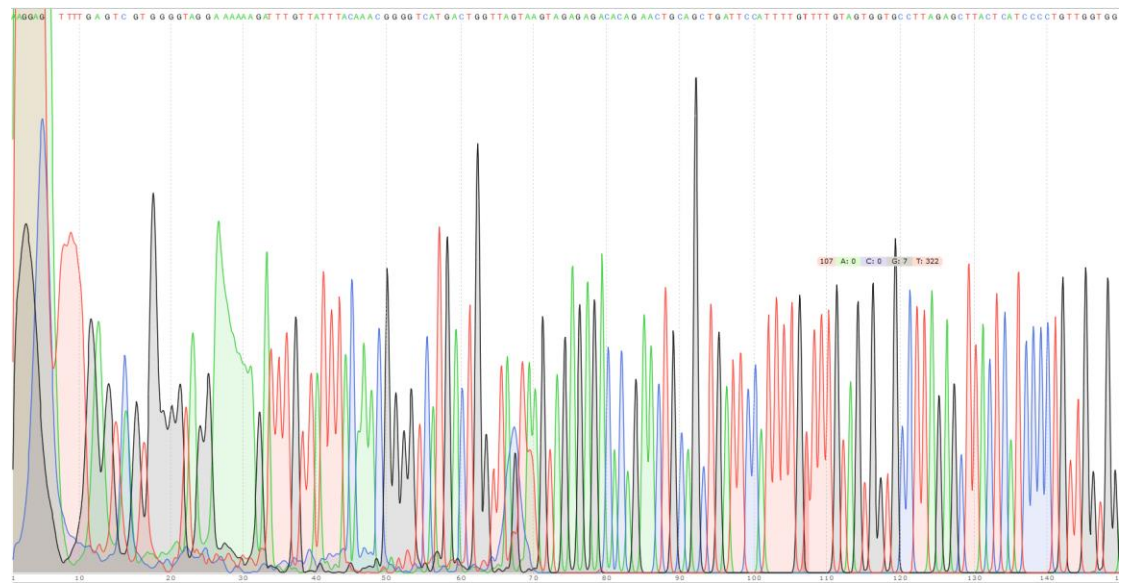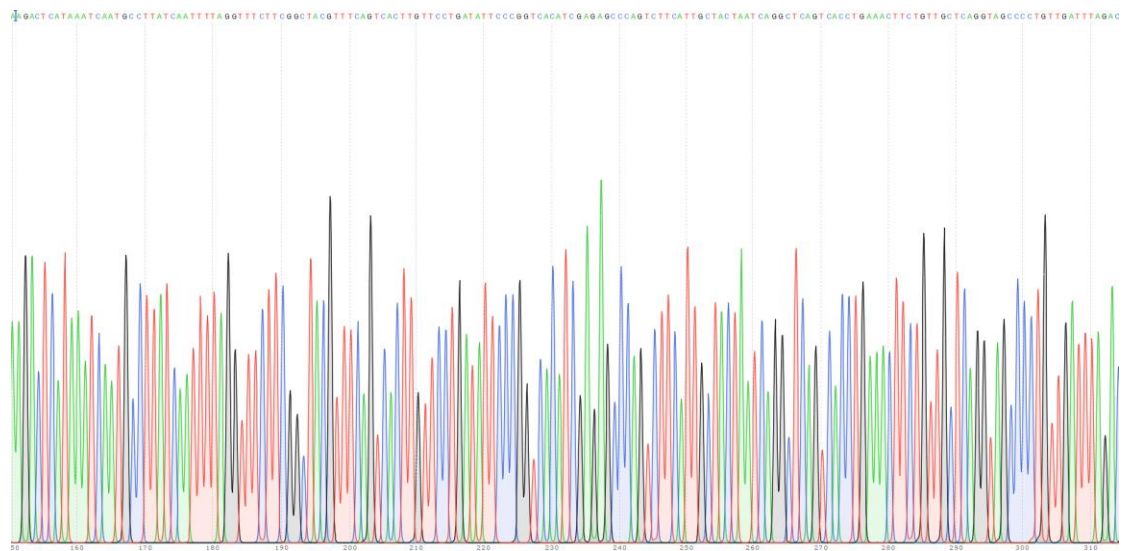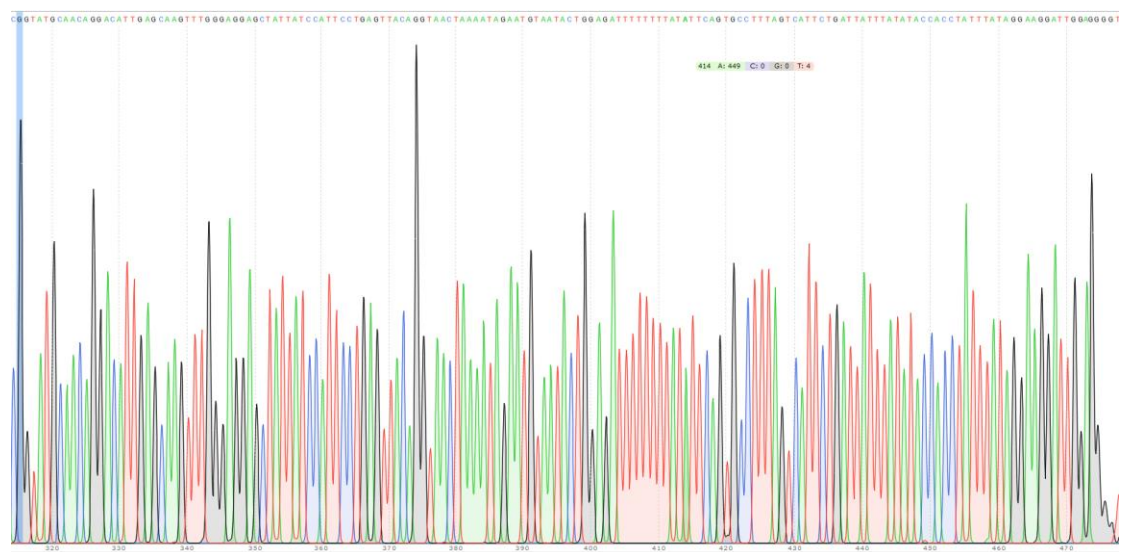

Supplement: Supplementary file 5 — Supplementary Material 5 [file 10020_2023_735_MOESM5_ESM.pdf]
