# Supplementary material for: Vitexin attenuates chronic kidney disease by inhibiting renal tubular epithelial cell ferroptosis via NRF2 activation
Source: Mol Med. 2023 Oct 27;29:147. doi: 10.1186/s10020-023-00735-1 (PMC10612207; doi:10.1186/s10020-023-00735-1)
Supplement: Supplementary file 6 — Supplementary Material 6 [file 10020_2023_735_MOESM6_ESM.pdf]

Supplementary table 1: Specific primers for qRT-PCR and guide RNA sequences

Species	Gene Name	Sequence (5’-3’)
Mouse	IL-1b	F: ACTGTGAAATGCCACCTTTTG
		R: TGTTGATGTGCTGCTGTGAG
	IL-6	F: ACAAAGCCAGAGTCCTTCAGAGAG
		R: TTGGATGGTCTTGGTCCTTAGCCA
	KIM-1	F: ACATATCGTGGAATCACAACGAC
		R: ACTGCTCTTCTGATAGGTGACA
	NGAL	F: GCAGGTGGTACGTTGTGGG
		R: CTCTTGTAGCTCATAGATGGTGC
	TNF-a	F: TCCCCAAAGGGATGAGAAG
		R: CACTTGGTGGTTTGCTACGA
	MCP-1	F: GCTCTCTCTTCCTCCACCAC
		R: ACAGCTTCTTTGGGACACCT
	GPX4	F: TGTGCATCCCGCGATGATT
		R: CCCTGTACTTATCCAGGCAGA
	ACSL4	F: CTCACCATTATATTGCTGCCTGT
		R: TCTCTTTGCCATAGCGTTTTTCT
	PTGS2	F: TTCAACACACTCTATCACTGGC
		R: AGAAGCGTTTGCGGTACTCAT
	HO-1	F: AAGCCGAGAATGCTGAGTTCA
		R: GCCGTGTAGATATGGTACAAGGA
	β-actin	F: GAGACCTTCAACACCCCAGC
		R: ATGTCACGCACGATTTCCC
Rat	Si-NRF2	Sense Sequence：CCUGAUCAGGCUCAGUCACUCGAUA
		Antisense Sequence：UAUCGAGUGACUGAGCCUGAUCAGG
Human	HO-1	F: AAGACTGCGTTCCTGCTCAAC
		R: AAAGCCCTACAGCAACTGTCG
	β-actin	F: CATGTACGTTGCTATCCAGGC
		R: CTCCTTAATGTCACGCACGAT
	gRNA target NRF2	F:CACCGTCGATGTGACCGGGAATATC
		R: AAACGATATTCCCGGTCACATCGAC

Supplementary table 2: Antibodies for western blotting

Proteins	Primary Antibodies	Secondary Antibodies
FN	1:1,000, ab2413 (Abcam)	HRP-Anti-Rabbit IgG, A0208 (Beyotime)
α-SMA	1:1,000, 19245 (CST)	HRP-Anti-Rabbit IgG, A0208 (Beyotime)
KIM-1	1:1,000, AF1817 (R&D Systems)	HRP-Anti-Goat IgG, A0108 (Beyotime)
NGAL	1:1,000, ab63929 (Abcam)	HRP-Anti-Rabbit IgG, A0208 (Beyotime)
NRF2	1:1,000, 16396-1-AP (Proteintech)	HRP-Anti-Rabbit IgG, A0208 (Beyotime)
MPO	1:1,000, AF3667 (R&D Systems)	HRP-Anti-Goat IgG, A0108 (Beyotime)
GPX4	1:1,000, 14432-1-AP (Proteintech)	HRP-Anti-Rabbit IgG, A0208 (Beyotime)
LaminB1	1:1,000, 12987-1-AP (Proteintech)	HRP-Anti-Rabbit IgG, A0208 (Beyotime)
GAPDH	1:1,000, 60004-1-Ig (Proteintech)	HRP-Anti-Mouse IgG, A0216 (Beyotime)
β-ACTIN	1:1,000, 66009-1-Ig (Proteintech)	HRP-Anti-Mouse IgG, A0216 (Beyotime)
DHODH	1:1,000, 14877-1-AP (Proteintech)	HRP-Anti-Rabbit IgG, A0208 (Beyotime)
FSP1	1:1,000, 20886-1-AP (Proteintech)	HRP-Anti-Rabbit IgG, A0208 (Beyotime)

Supplementary table 3: Antibodies used in the IF

Proteins	Primary Antibodies	Secondary Antibodies
Col1A1	1: 150, 72026 (CST)	Donkey anti-Rabbit IgG (H+L) Highly Cross-Adsorbed Secondary Antibody, Alexa Fluor 488, Thermo Fisher Scientific, #21206
NRF2	1:100, 16396-1-AP (Proteintech)	Donkey anti-Rabbit IgG (H+L) Highly Cross-Adsorbed Secondary Antibody, Alexa Fluor 488, Thermo Fisher Scientific, #21206

Supplementary table 4: Antibodies applied in the IHC

Proteins	Primary Antibodies	Secondary Antibodies
F4/80	1:100, 70076 (Cell Signaling Technology)	ZLI-9018 (ZSGB-BIO)
GPX4	1:100, 14432-1-AP (Proteintech)	ZLI-9018 (ZSGB-BIO)
4-HNE	1:100, MAB3249-SP (R&D Systems)	ZLI-9018 (ZSGB-BIO)
NRF2	1:100, 16396-1-AP (Proteintech)	ZLI-9018 (ZSGB-BIO)
