# Supplementary material for: Vitexin attenuates chronic kidney disease by inhibiting renal tubular epithelial cell ferroptosis via NRF2 activation
Source: Mol Med. 2023 Oct 27;29:147. doi: 10.1186/s10020-023-00735-1 (PMC10612207; doi:10.1186/s10020-023-00735-1)
Supplement: Supplementary file 7 — Supplementary Material 7 [file 10020_2023_735_MOESM7_ESM.docx]

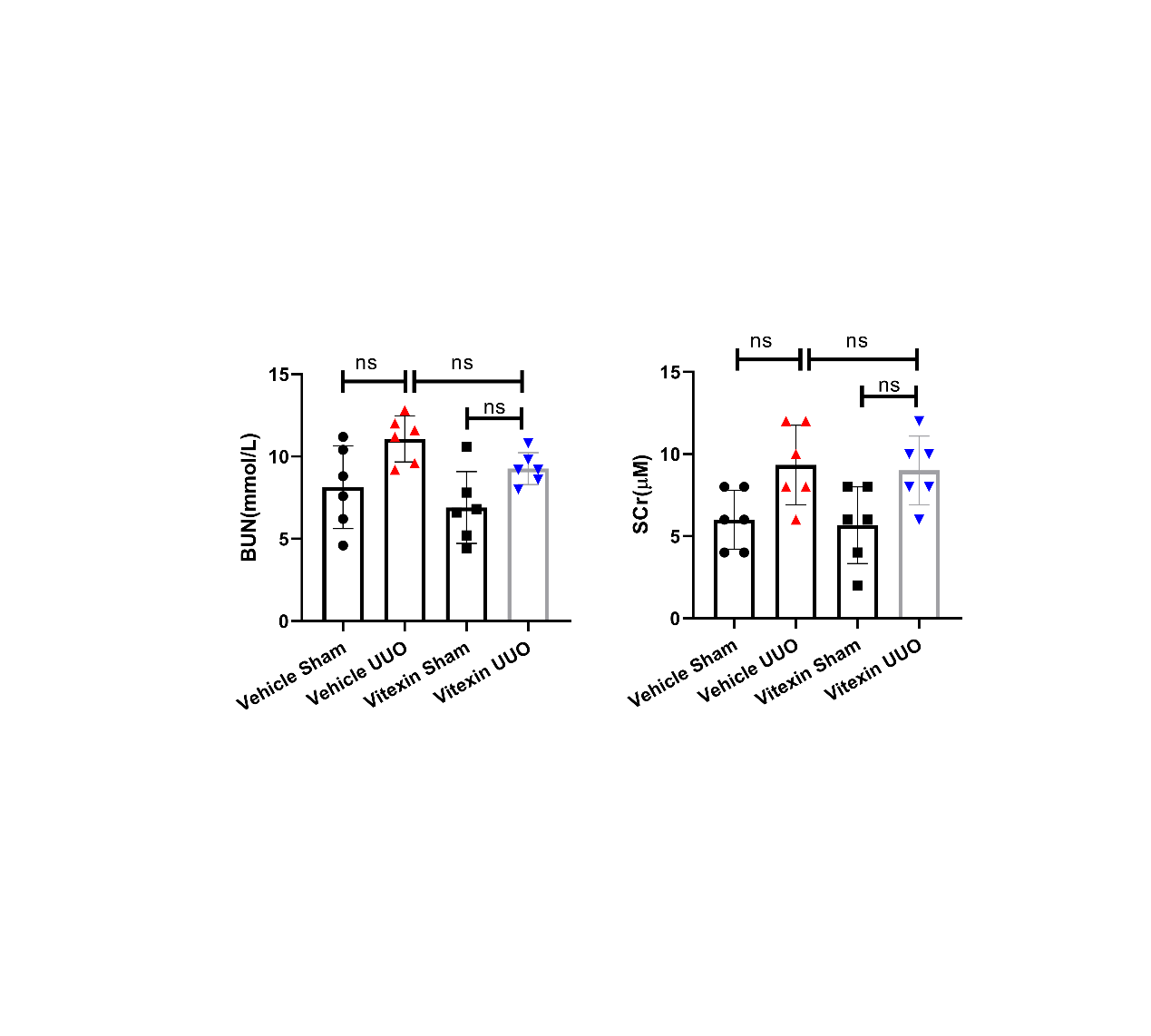


**Supplementary Fig. 1**. The levels of blood urea nitrogen (BUN) and serum creatinine (Scr) in UUO mice treated with or without vitexin. All data are shown as the mean ± S.D (n= 6). *ns: no significance (one-way ANOVA).*


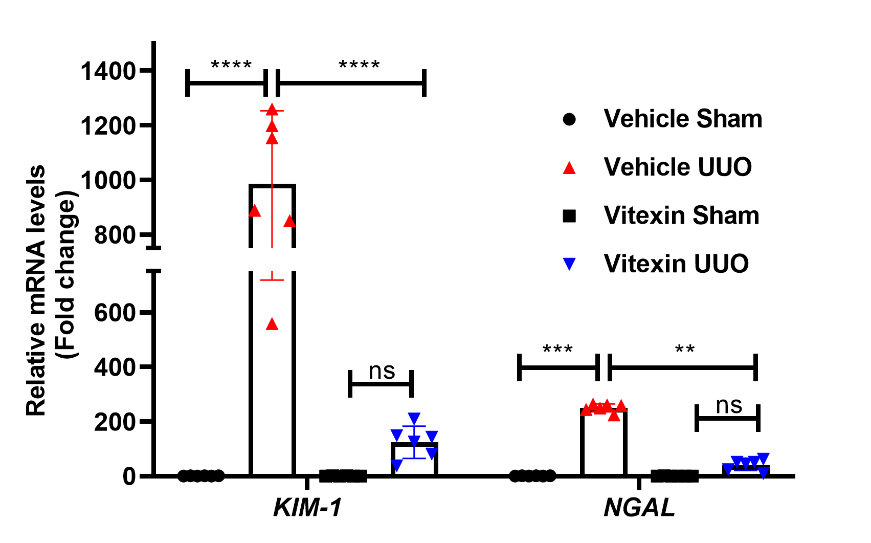


**Supplementary Fig. 2**. The mRNA levels of renal KIM-1 and NGAL in kidneys of UUO mice were analyzed by qRT‒PCR. The results are shown as the mean ± S.D. of 6 mice in each group. *****P<0·0001, ***P<0·001, **P<0·01, ns: no significance (two-way ANOVA).*


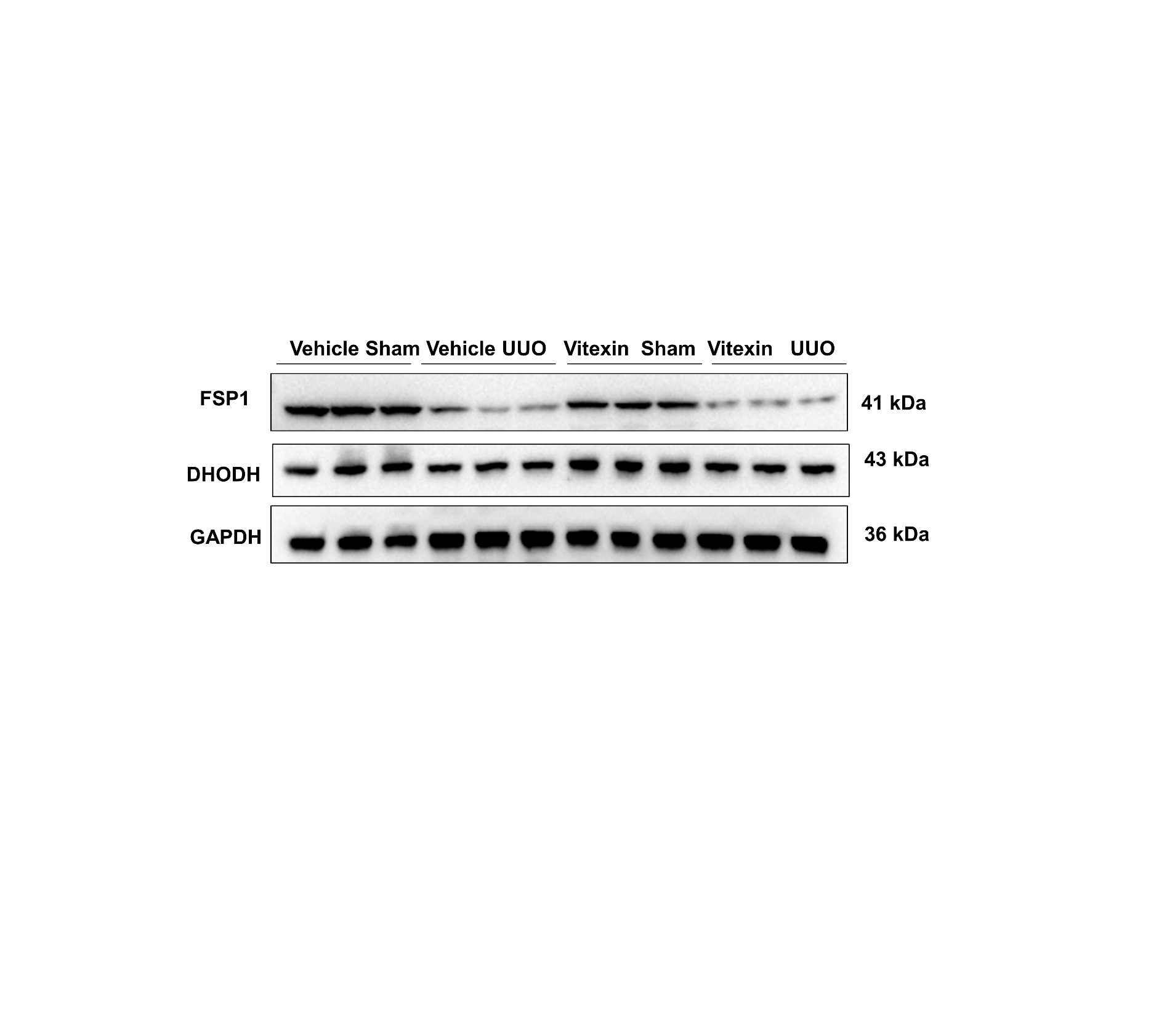


**Supplementary Fig. 3.** The protein expression of FSP1 and DHODH in renal tissues was detected by western blot.


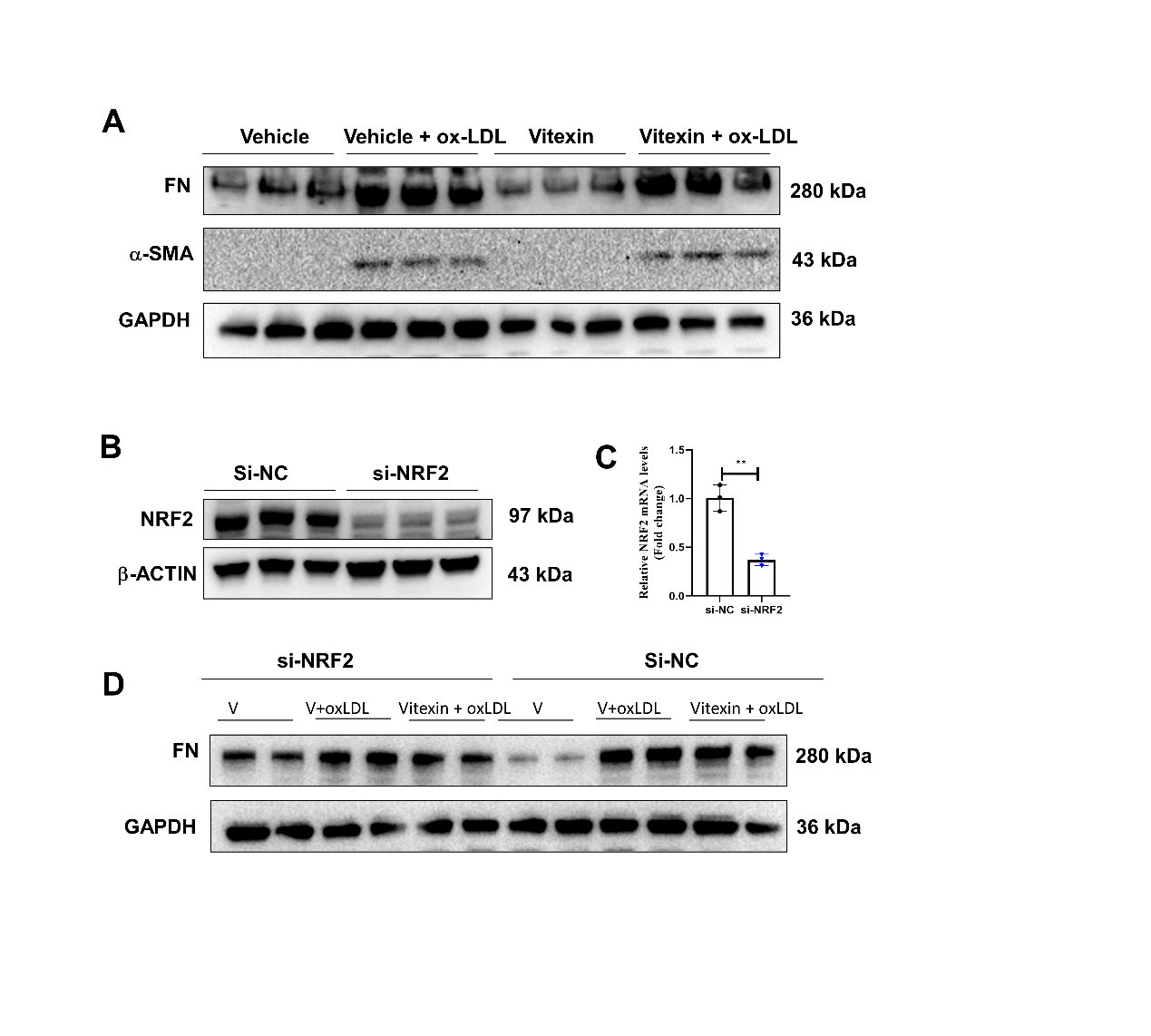


**Supplementary Fig. 4.** (A) Western blot images showing the protein level of α-SMA and FN in NRK-49F cells treated with ox-LDL (5 μg/ml for 24 h) with or without vitexin (100 μM) treatment. Knock down the expression of NRF2 by siRNA in NRK-49F cells was analyzed by western blot (B) and qRT-PCR (C). (D) Western blot images showing the protein level of FN in NRF2 knock down or NC (negative control) NRK-49F cells treated with ox-LDL (5 μg/ml for 24 h) with or without vitexin (100 μM) treatment.


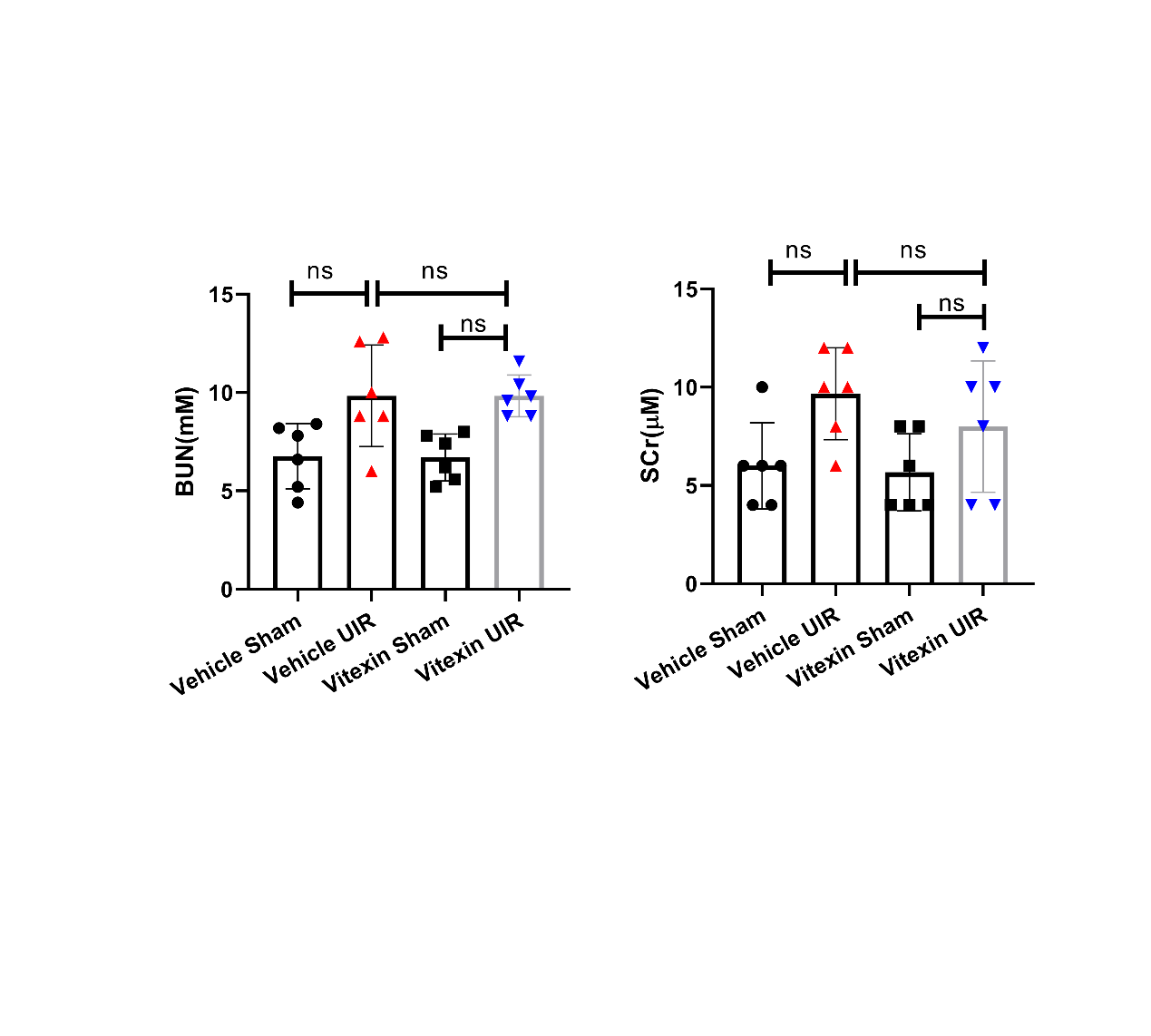


**Supplementary Fig. 5.** The levels of blood urea nitrogen (BUN) and serum creatinine (Scr) in UUO mice treated with or without vitexin. All data are shown as the mean ± S.D (n= 6). *ns: no significance (one-way ANOVA).*
